# Triangulating evidence for the causal impact of single-intervention zinc supplement on glycaemic control for type 2 diabetes: systematic review and meta-analysis of randomised controlled trial and two-sample Mendelian randomisation

**DOI:** 10.1017/S0007114522002616

**Published:** 2023-06-14

**Authors:** Zhiyang Wang, Carine Ronsmans, Benjamin Woolf

**Affiliations:** 1 Faculty of Epidemiology and Population Health, London School of Hygiene and Tropical Medicine, London, UK; 2 Institute for Molecular Medicine, University of Helsinki, Helsinki, 00290, Finland; 3 Medical Research Council Integrative Epidemiology Unit, University of Bristol, Bristol, UK; 4 Department of Psychological Science, University of Bristol, Bristol, UK

**Keywords:** Zn, Type 2 diabetes, Mendelian randomisation, Meta-analysis

## Abstract

Although previous studies suggested the protective effect of Zn for type 2 diabetes (T2D), the unitary causal effect remains inconclusive. We investigated the causal effect of Zn as a single intervention on glycaemic control for T2D, using a systematic review of randomised controlled trials and two-sample Mendelian randomisation (MR). Four primary outcomes were identified: fasting blood glucose/fasting glucose, HbA1c, homeostatic model assessment for insulin resistance (HOMA-IR) and serum insulin/fasting insulin level. In the systematic review, four databases were searched until June 2021. Studies, in which participants had T2D and intervention did not comprise another co-supplement, were included. Results were synthesised through the random-effects meta-analysis. In the two-sample MR, we used single-nucleotide polymorphisms (SNP) from MR-base, strongly related to Zn supplements, to infer the relationship causally, but not specified T2D. In the systematic review and meta-analysis, fourteen trials were included with overall 897 participants initially. The Zn supplement led to a significant reduction in the post-trial mean of fasting blood glucose (mean difference (MD): −26·52 mg/dl, 95 % CI (−35·13, −17·91)), HbA1c (MD: −0·52 %, 95 % CI: (−0·90, −0·13)) and HOMA-IR (MD: −1·65, 95 % CI (−2·62, −0·68)), compared to the control group. In the two-sample MR, Zn supplement with two SNP reduced the fasting glucose (inverse-variance weighted coefficient: −2·04 mmol/l, 95 % CI (−3·26, −0·83)). From the two methods, Zn supplementation alone may causally improve glycaemic control among T2D patients. The findings are limited by power from the small number of studies and SNP included in the systematic review and two-sample MR analysis, respectively.

Type 2 diabetes (T2D) is a chronic disease that causes a patient’s body not to respond normally to insulin called insulin resistance. Impaired insulin sensitivity reduces both the absorption and reserve of insulin in organs. At an early stage, the patient’s serum insulin levels will increase to compensate for the increased insulin resistance. However, serum insulin will eventually decrease and blood sugar levels rise^(1,2)^. Diabetes symptoms include polydipsia, fatigue and unintended weight loss and cause lower self-evaluation of physical and mental health^(3)^. Globally, the age-standardised incidence rate increased by 3·23 % to 279·13 per one million from 2007 to 2017^(4)^. It brings multiple challenges to medicine, primary care and the economy. Four-fifths of diabetes patients currently live in low- and middle-income countries, and diabetes increase their susceptibility to other infections such as hepatitis B^(5)^. In India, nearly half of patients with T2D did not receive a proper diagnosis and are at risk of hidden complications as the disease progresses^(6)^. Moreover, patients faced substantial cost-of-illness and lower employment chances, while government health expenditure on T2D is projected to decline^(7,8)^. It is therefore critical to find a cost-effective and accessible intervention to address the threat of T2D. Zn, as a usual commercial supplement, could be a good candidate solution.

Zn supplementation was demonstrated to have a protective effect on insulin and metabolism in the hyperglycaemic environment in animal models^(9–11)^. The Zn ion plays an essential role with insulin in the pancreatic *β* cell, activating multiple cell signalling cascades^(12)^. Zn ions coordinate six insulin monomers (hexamerisation), which enhance the stability of insulin and the storage capacity of the insulin-secreting vesicles^(13–15)^. Moreover, Zn is known for its antioxidative property as a cofactor of the important antioxidative enzyme, which may reduce lipid peroxidation and development of insulin resistance in diabetes mellitus^(16–18)^.

Randomised controlled trials (RCT) have evaluated the impact of Zn or co-supplements on various types and progression of diabetes^(19,20)^. One meta-analysis, published in 2019, found that Zn supplements in both single factor and co-supplements significantly reduced glycaemic indicators such as fasting glucose^(21)^. A 2021 meta-analysis suggested that low-dose and long-duration single Zn supplements had a beneficial impact on many T2D and cardiovascular disease risk factors^(22)^. The multi–nutrient intervention in the previous review included the interactions between nutrients, so it may not provide a valid estimate of Zn ’s effect as a single supplement. For example, Fe and Ca supplements may interfere with the repletion of Zn and inhibit Zn’s bioaccessibility, and the interaction mechanism within more micronutrients remained complicated^(23,24)^. Neither study was limited to a specific type of diabetes and even included healthy participants at high risk. This could mask the real effect of the single Zn supplement on prevalent T2D patients.

Mendelian randomisation (MR) is another study design that could help us to evaluate the causality between single Zn supplements and diabetes. MR is regarded as a ‘natural experiment’, which leverages the random inheritance of genetic variants to approximate the random allocation of the participants to a modifiable exposure, which the variants are robustly associated with^(25–28)^. Because genes are inherited at birth, MR estimates are interpreted as the lifetime effect of the exposure, while RCT studies can only provide the effects over shorter periods of time. Single-nucleotide polymorphisms (SNP) are a one-row change in the DNA double helix which occurs in at least 5 % of the population, which are the most common type of genetic variant. The associations between SNP and phenotypes are generally detected through genome-wide association studies (GWAS). These studies involve a hypothesis-free test of association between every measured SNP and the phenotype. To prevent false positives, stringent multiple testing criteria are applied when deciding if an SNP is ‘genome-wide significant’. By measuring the whole genome, it is, therefore, possible to characterise the association of SNP with exposure and outcomes, such as metabolic response to a specific diet^(29–31)^. Nevertheless, because GWAS are hypothesis-free, they do not provide insight into the mechanism linking the SNP with an associated phenotype-like Zn supplementation. It is, however, unlikely that any associated SNP would be a ‘gene for zinc supplementation’. Instead, they would likely be SNP that influence risk factors for taking Zn supplementation (such as a generic propensity to health-seeking behavior), or SNP that happen to have a different prevalence in subgroups who are more likely to take the supplements (residual population structure).

MR has been used to obtain an unbiased assessment of nutritional status to the outcome of interest. Researchers notably demonstrated the association of long-term testosterone exposure with health outcomes by MR^(32)^. Collaborating with RCT, MR could help to strengthen the causal estimation, particularly in cumulating exposure inside the body^(33)^, which may provide suggestions for health professionals to give patients accessible supplements for diabetes management, especially under the source-limited condition. In this study, we aimed to systematically assess the unmasked effect and possible causal inference about the association between single Zn supplements and glycaemic control among T2D patients, using a systematic review of RCT and two-sample MR.

## Method

### Systematic review

#### Protocol and ethnics

This meta-analysis followed the Preferred Reporting Items for Systematic Reviews and Meta-analyses (PRISMA) reporting guideline^(34)^. This study received ethics approval from the London School of Hygiene and Tropical Medicine (LSHTM) MSc Research Ethics Committee (reference: 25 896).

#### Eligibility criteria

The study eligibility criteria were specified using Population, Intervention, Comparison, Outcomes and Study (PICOS) frame^(34)^.

##### Inclusion criteria

- Study population: Any human participants with T2D. There was no restriction on the demographic characteristics.
- Intervention: Zn administration as the single supplement intervention.
- Comparison: Use of placebo.
- Outcome: Primary outcomes included fasting blood glucose, HbA1c, homeostatic model assessment for insulin resistance (HOMA-IR) and serum insulin level. Secondary outcomes included any other quantitative outcomes related to diabetes control.
- Study design: Any RCT evaluating the association between Zn intervention and glycaemic control.

##### Exclusion criteria

- Full text was not available or accessible.
- Co-supplement with other nutrition would not be included to concentrate on the Zn-only effect.

#### Information sources and search strategy

PubMed, CINAHL Plus, EMBASE and Web of Science were searched from the establishment of each database (in 1996, 1937, 1961 and 1997, respectively) to June 2021. We also checked the references and citations of all included studies for eligibility criteria. The search strategy was developed by and modified from the previous systematic review^(21)^. The terms were structured according to each database‘s guidelines and searched by titles and abstracts. The detail is presented in supplementary method 1.

#### Selection process

The abstracts and titles were screened by the eligibility criteria. The first unblinded reviewer screened all the citations and the second one checked a random 10 % sample of all citations due to the limitation of human resources. The percentage agreement and the Gwet AC (agreement change adjusted) between the two reviewers were calculated to test the interrater reliability^(35)^.

#### Data extraction and item

The standard form for intervention reviews for RCT developed by the Cochrane Collaboration was used to exact the data^(36)^. The extraction process was conducted twice to minimise bias or entry error. The specific data items are shown below:

- Eligibility criteria: inclusion and exclusion criteria
- Study design (parallel or crossover), assignment of each arm and analysis plan (intention-to-treat or per-protocol)
- Participants: the total number of randomised and analysed participants, numbers in each arm (pre-trial and post-trial), geographic information and demographic characteristics.
- Intervention and control arms: formulation and dosage of Zn, administration method, description of the control arm (placebo) and study duration.
- Results: the measure of effect (means, mean difference or change score), standard error of effect measure, statistical significance and any other results such as OR.
- Information about risk-of-bias: any information causing bias, such as missing participants.

#### Effect measure

The primary measure was the post-trial mean difference (MD) with standard error (se) for each outcome. The change scores are the change of mean from pre-trial to post-trial in each arm so that the difference of change scores between Zn intervention and control was our secondary effect measure. We either converted units or used the standardised mean difference (SMD) to harmonise the measurement scales^(37)^.

#### Synthesis methods

The meta-analyses were conducted to synthesise the results. The subgroup analyses were stratified by studies that clearly stated the participants were T2D patients and that did not specify the participant’s diabetes type.

The Cochran-Q *χ* ^2^ test and the *I* ^2^ index were used to assess the statistical heterogeneity. We also examined previous systematic reviews for their findings on heterogeneity. If there was no statistically significant heterogeneity (*P* > 0·05 and *I* ^2^ < 40 %) or evidence about existing heterogeneity from previous studies, a pooled effect would have been calculated with a fixed-effects model, which assumed that the observed differences among study results are due solely to chance^(37)^. If there was significant heterogeneity, the summary estimate and confidence/prediction interval would come from the random-effects model, which assumed that the different studies are estimated differently, then providing the average intervention effect^(37)^.

If the study included more than two intervention arms, we reviewed only interventions that met the eligibility criteria or summarised at the arm level. We only interpreted studies that were judged as low risk of overall bias in the main result section to avoid biases.

#### Reporting bias assessment

If there were five or more studies in the meta-analysis, we assessed publication bias by graphical methods (funnel plots, which indicate the potential presence of reporting biases)^(38)^. In the plots, the x-axis represents the effect estimate, and the y-axis represents the standard error of the effect estimate. If there was publication bias, it would lead to an asymmetrical appearance of the plot^(39)^. Furthermore, the Egger test was used for reporting bias to evaluate the small study effect^(40)^.

#### Additional analysis

If the heterogeneity was significant in the meta-analysis, the linear random-effect meta-regression was used to explore the trial-level covariates that contributed to the heterogeneity^(41)^. The candidate covariates were Zn dose, trial duration and whether the trial specified the participant’s diabetes type (T2D, not specified types). The first two were treated as continuous variables and the third was a binary variable.

We illustrated the effect of interest if only one study reported any secondary outcomes. If more than one study reported a type of secondary outcome, a meta-analysis was conducted regardless of the risk-of-bias. We also performed a sensitivity analysis which included all trials into meta-analysis regardless of risk assessment for comparing the results to evaluate the impact of bias.

### Two-sample Mendelian randomisation

This two-sample MR study followed STROBE-MR Guidelines^(42)^.

#### Study design and data sources

Sample sizes and GWAS data including both exposure and outcome were utilised from the MR-base, which is a repository of GWAS summary statistics^(43)^. We included all ‘zinc supplement’ SNP (Open GWAS ID: ukb-b-10567, ukb-b-13891, ukb-a-496) that meet the p-value threshold of <5 × 10^−8^ from the MR-base GWAS catalog. We also extracted the summary data (*β* and se) for the diabetes outcomes: fasting blood glucose (Open GWAS ID: ieu-b-114, GCST000568, ebi-a-GCST007858, ebi-a-GCST005186, ieu-b-113), HbA1c (Open GWAS ID: ieu-b-103, ieu-b-104), HOMA-IR (Open GWAS ID: ieu-b-118, ebi-a-GCST005179) and fasting insulin (Open GWAS ID: ebi-a-GCST005185, ebi-a-GCST000571, ebi-a-GCST007857, ieu-b-115, ieu-b-116). Description of the methods for these GWAS can be found in reference^(44–46)^.

We performed clumping using the genetic distance of 10 000 kilobases and an linkage disequilibrium *R* ^2^ of 0·001 from the 1000 genomes European reference sample. When SNP were not measured in the outcome data set, we used proxy SNP with a minimum linkage disequilibrium *R* ^2^ of 0·8^(47)^. Palindromic SNP were imputed if they had a minor allele frequency at <0·3^(48,49)^. We allowed MR-base to automatically harmonise the exposure and outcome data and attempt to align palindromic SNP based on their minor allele frequency.

#### Assumptions and assessment

There are three routine assumptions and two additional assumptions in two-sample MR.

1. Relevance: The genetic variants are associated with the exposure of interest.
  - This assumption was assessed via F statistic for the gene-exposure relationship.
2. Independence: The genetic variants share no unmeasured cause with the outcome.
  - This assumption was by ensuring that the GWAS have adequately controlled for plausible confounders of the gene-outcome association by adjusting for at least ten principal components or using a linear mixed model.
3. Exclusion restriction: The genetic variants do not affect the outcome except through their potential effect on the exposure of interest.
  - Pleiotropy can violate this assumption when genes influence two or more traits. Several secondary analyses were used to detect and adjust the pleiotropy such as MR-Egger regression, weighted median and weighted mode analysis.
4. The samples for exposure and outcome assessment are independent.
  - We excluded the outcome summary data which had the same consortium as the exposure data to meet this assumption.
5. The samples for exposure and outcome assessment are from the same population.
  - We chose the same outcome population category such as European with the exposure population, ensuring the fifth assumption.

#### Statistical methods

We used the ‘TwoSampleMR’ package in the R environment (version 4.1.0) to conduct the two-sample MR analyses^(43)^. This package supported the harmonisation process to ensure that both SNP were coded from the same strand when SNP were palindromic^(48)^. We used the inverse variance weighting estimator to evaluate the causal inference. It meta-analyses the Wald ratios which is the ratio of the SNP outcome association to SNP exposure association. Due to the difference in MR-base, two outcomes were slightly changed compared with the systematic review: fasting glucose and fasting insulin.

Cochran Q statistics can be used to assess evidence of heterogeneity in consideration of pleiotropy^(50)^. The statistically significant level was at the *P* < 0·05. Steiger filtering checked the direction of causation between the exposure and outcome for each SNP^(51)^.

We, therefore, used the exposure of Ca supplement and outcome of hair colour as the negative controls to detect the potential residual bias due to nutrients supplementation and population structure, respectively. These were chosen because Ca supplementation is a type of nutritional supplement which is not thought to impact diabetes, and hair colour is known to vary with population structure within the UK but is unlay to have any true causal association with Zn supplementation.

## Results

### Systematic review

#### Study selection

**Fig. 1.**
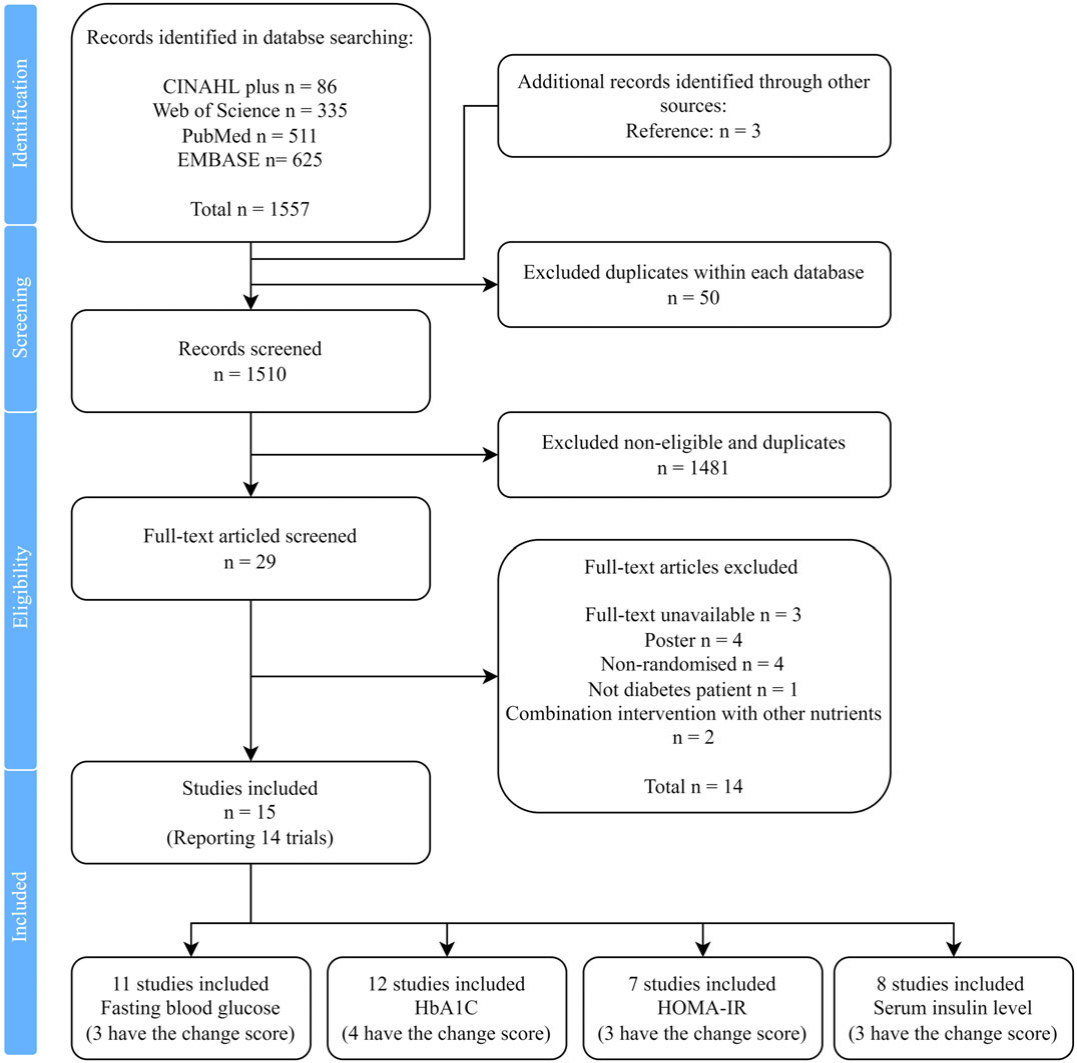
PRISMA flow diagram of information through the different phases of the systematic review.

A total of fifteen studies, reporting on fourteen trials, were identified for inclusion in the review (Fig. 1). One trial generated two studies (Parham *et al.* ^(62)^ and Heidarian *et al.* ^(63)^). From the four databases, 1557 citations were found, and three studies were identified by reference reading. 1531 citations were removed because of duplication or unfulfilling inclusion criteria with title or abstract. After the full-text screening of twenty-nine studies, fourteen studies were excluded because they did not meet the criteria. There were 11, 12, 7 and 8 studies measuring fasting blood glucose, HbA1c, HOMA-IR and serum insulin levels, respectively.

The percentage agreement was 96·32 % and Gwet AC was 0·9619 between two reviewers, with strong evidence of significance (*P* < 0·05), which indicated a very good inter-reviewer reliability for the selection process. No unpublished relevant studies were obtained.

#### Study characteristics

Thirteen trials used a parallel design and one used a cross-over design (Parham *et al*.^(62)^ and Heidarian *et al*.^(63)^). Details of each trial are presented in Table 1. A total of 897 participants were randomly allocated at initial, and all trials had a relatively small sample size. The date of publication ranged from 2003 to 2021. Most trials claimed that there was no significant imbalance (*n*=7) in age and sex, or they used matched methods (*n*=2). One trial had adolescent participants, while the other’s average ages were above 45-year-old. Four trials did not specify the type of diabetes in the inclusion criteria and patients had complications: diabetic foot ulcer^(59)^, diabetic haemodialysis with Zn deficiency^(56)^, diabetic retinopathy^(60)^ and β-thalassemia major complicated^(58)^.

**Table 1.** Summary of included studies evaluating the effect of Zn intervention in the systematic review

Study	RCTdesign	Country	Randomisation participants,*n* control/intervention*	Duration,month	Sex,male/female	Age (Mean), year†	Age (Standard deviation), year	Formulation/elemental Zn dose,mg/d	Health status
Afkhami and Ardekani, 2008^(52)^	Parallel	Iran	40 (total)	1·5	16/24	52·67	8·60	Zn sulfate/149·82	Type 2 diabetes
Asghari, 2019^(53)^	Parallel	Iran	30/30	3	32/28	45·5 *v*. 46·2	5·4 *v*. 5·3	Zn gluconate/4·29	Type 2 diabetes
Burki, 2017^(54)^	Parallel	Pakistan	46/54	3	NA	NA		Zn sulphate/4·54§	Type 2 diabetes
Gunasekara, 201^(55)^ ‡	Parallel	Sri Lanka	31/29	4	23/27	51·2 *v*. 54·1	6·0 *v*. 6·0	Elemental Zn/22‖	Type 2 diabetes
Hosseini, 2021^(56)^	Parallel	Iran	25/21	2	22/24	54·1 *v*. 55·6	5·4 *v*. 7·3	Elemental Zn/50	Diabetic haemodialysis patients with Zn deficiency
Khan, 2013^(57)^	Parallel	India	27/27	3	NA	56·0 *v*. 56·3	6·86 *v*. 6·6	Zn sulphate/11·35	Type 2 diabetes
Matter, 2020^(58)^	Parallel	Egypt	40/40	3	38/42	16·1 *v*. 16·4	1·4 *v*. 1·4	Zn gluconate/7·72	Diabetes with β-thalassemia major complicated
Momen-Heravi, 2017^(59)^	Parallel	Iran	30/30	3	42/18	60·0 *v*. 58·3	10·0 *v*. 8·6	elemental Zn/50	Diabetes with diabetic foot ulcer
Naghizadeh, 2018^(60)^	Parallel	Iran	22/23	3	20/25	58·0 *v*. 57·4	5·9 *v*. 5·8	Zn gluconate/4·29	Patients with diabetic retinopathy
Nazem, 2019^(61)^	Parallel	Iran	35/35	2	38/32	54·3 *v*. 53·3	7·2 *v*. 7·4	Zn gluconate/7·15	Type 2 diabetes
Parham, 2008^(62)^, Heidarian, 2009^(63)^	Cross-over	Iran	42 (total)	2·5	24/18	52·0 54·5	9·3 *v*. 9·2	elemental Zn /30	Type 2 diabetes
Pérez, 2018^(64)^	Parallel	Chile	15/13	12	13/15	30–65		elemental Zn /6·81	Type 2 diabetes with further selection criteria
Roussel, 2003^(65)^	Parallel	Tunis	29/27	6	NA	55·5 *v*. 51·5	1·4 *v*. 1·6	Zn gluconate/4·29	Type 2 diabetes
Witwit, 2021^(66)^	Parallel	Iraq	25/25	1·5	NA	25–60		Zn sulphate/11·35	Type 2 diabetes

RCT, randomised controlled trial.

* smaller size of participants due to lost during the trial

† Values are mean (control, intervention) or inclusion range.

‡ It was a three-arm study, and we only extracted Zn and its control arm.

§ Oral hypoglycaemic arm and Zn plus oral hypoglycaemic arm.

‖ Multivitamin/mineral arm and Zn plus multivitamin/mineral arm.

The duration of Zn supplementation ranged from 1·5 to 12 months, with a mean and median duration of 3·53 months and 3 months. Three trials contained basic supplements care besides the Zn administration and placebo: oral hypoglycaemic agent (in Burki *et al.* ^(54)^ and Khan *et al*.^(57)^) and multivitamin/mineral (in Gunasekara *et al*.^(55)^). Gunasekara *et al.* ^(55)^ had three arms and we only extracted the information of the Zn+ multivitamin/mineral (intervention) and multivitamin/mineral group (control). Each dose of Zn gluconate and Zn sulfate was multiplied by 0·143 or 0·227 to obtain the appropriate dose of elemental Zn^(22)^. The mean dose of elemental Zn included in these interventions was 25·83 mg/d (range: 4·29–149·82 mg/d; median: 9·25 mg/d).

#### Results of individual studies

Nine trials measured post-trial means with se of fasting blood glucose as outcomes. Seven of the nine trials were at low risk of bias. Eight trials including the cross-over trial measured post-trial HbA1c as an outcome. Six of the eight trials were at low risk of bias. Six trials with low risk-of-bias had eligible measured post-trial HOMA-IR scores. Six trials with low risk-of-bias had eligible measured post-trial serum insulin as an outcome. We standardised the measurement of serum insulin level on a single scale through Hedges’ g score and presented the SMD.

The detail of the risk of bias assessment for each study is presented in Supplementary result 1, Supplementary Fig. 1 and Supplementary Table 1.  

#### Synthesis of results

**Fig. 2.**
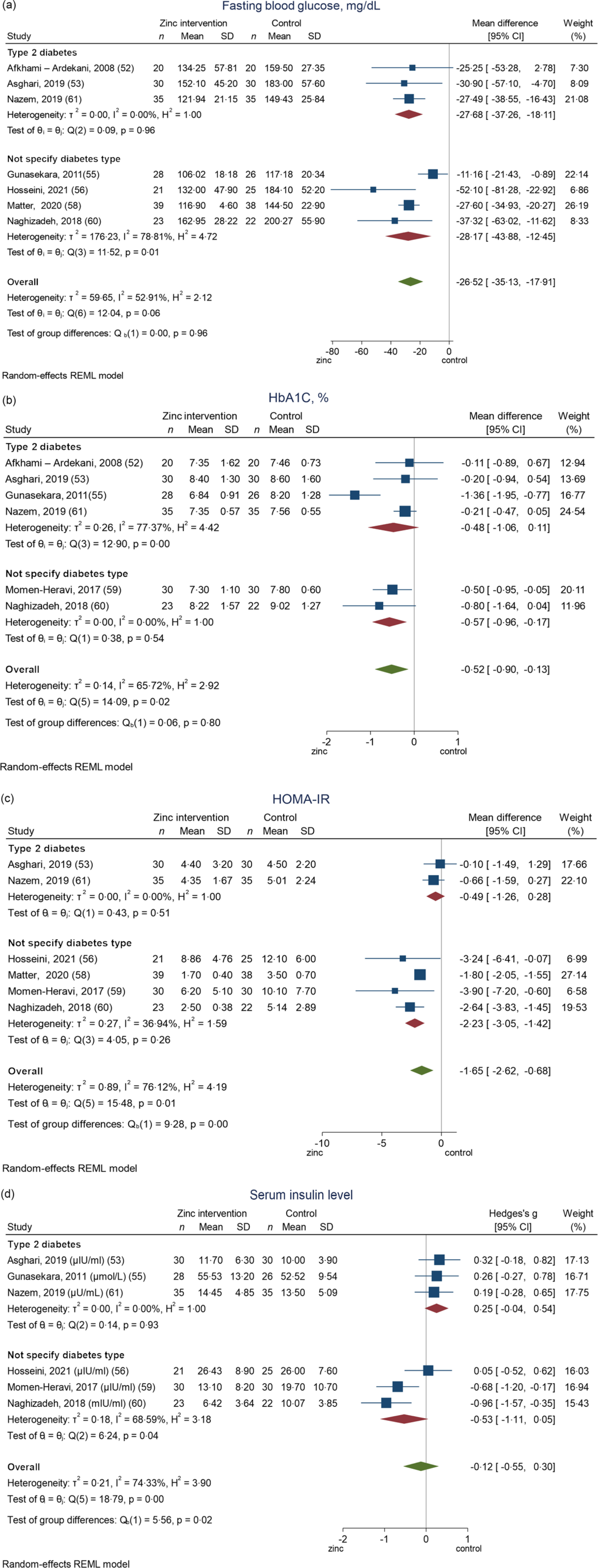
Forest plots summarising the MD or SMD of post-trial fasting blood glucose (a), HbA1c (b), HOMA-IR (c) and serum insulin level (d) between Zn intervention and control arms for low risk-of-bias trials. MD, mean difference; SMD, standardised mean difference; HOMA-IR, homeostatic model assessment for insulin resistance.

The random-effects model was used for meta-analysis for all outcomes, because of the *I*^2^ (>40 %), the Cochran-Q test (*P* < 0·05) in most outcomes and the findings of previous systematic reviews^(22)^. The meta-analyses including the only low risk-of-bias trials are presented in Fig. 2.

##### Fasting blood glucose

Among the T2D patients, the pooled MD was −27·68 mg/dl (95 % CI (−37·26, −18·11)), comparing Zn intervention to the control arm (Fig. 2(a)). Among the participants without specified diabetes type, the pooled MD was −28·17 mg/dl (95 % CI (−43·88, −12·45)). Both stratified analyses showed strong evidence of effect and the two types of trials were not significantly different (Test of group difference *P* = 0·96). The overall pooled MD showed that the Zn intervention had a significantly lower level of fasting blood glucose at the end of the trial, compared with the control arm (MD: −26·52, 95 % CI (−35·13, −17·91)). Regardless of the Risk-of-bias (ROB) of the trials, the overall pooled MD was −26·87 mg/dl (95 % CI (−35·74, −18·00)) (online Supplementary Fig. 2(a)).

##### HbA1C

T2D patients have a decrease (MD: −0·48, 95 % CI (−1·06, 0·11)) in HbA1c, but this difference was not significant between arms (Fig. 2(b)). Among the participants without specified type, the pooled MD was −0·57 % (95 % CI (−0·96, −0·17)) in HbA1c, compared to Zn intervention and control arm. There was no significant difference between those two types of trials (Test of group difference *P* = 0·80). Overall, there was a significant reduction in post-trial HbA1c percentage between Zn intervention and control at the end of the trial (MD: −0·52, 95 % CI (−0·90, −0·13)). The overall pooled MD that also included all trials regardless of the ROB was −0·64 % (95 % CI (−1·05, −0·22)) (online Supplementary Fig. 2(b)).

##### Homeostatic model assessment for insulin resistance

The pooled MD was −0·49 (95 % CI (−1·26, 0·28)) in T2D patients and −2·23 (95 % CI (−3·05, −1·42)) among patients with unspecified type in the post-trial HOMA-IR significantly (Fig. 2(c)). There was a significant difference detected between those two types of trials (Test of group difference *P* < 0·01). The pooled result shows that the Zn intervention arm had a 1·65 lower (95 % CI (−2·62, −0·68)) HOMA-IR score than the control arm with strong evidence.

##### Serum insulin level

For serum insulin level (Fig. 2(d)), the SMD was 0·25 (95 % CI (−0·04, 0·54)) in T2D patients and −0·53 (95 % CI (−1·11, 0·05)) among the participants without specified diabetes type, with only weak evidence. Overall, the pooled result shows that there was no significant SMD in serum insulin level between Zn intervention and control arms at the end of the trial (SMD: −0·12, 95 % CI (−0·55, 0·30)).

**Fig. 3.**
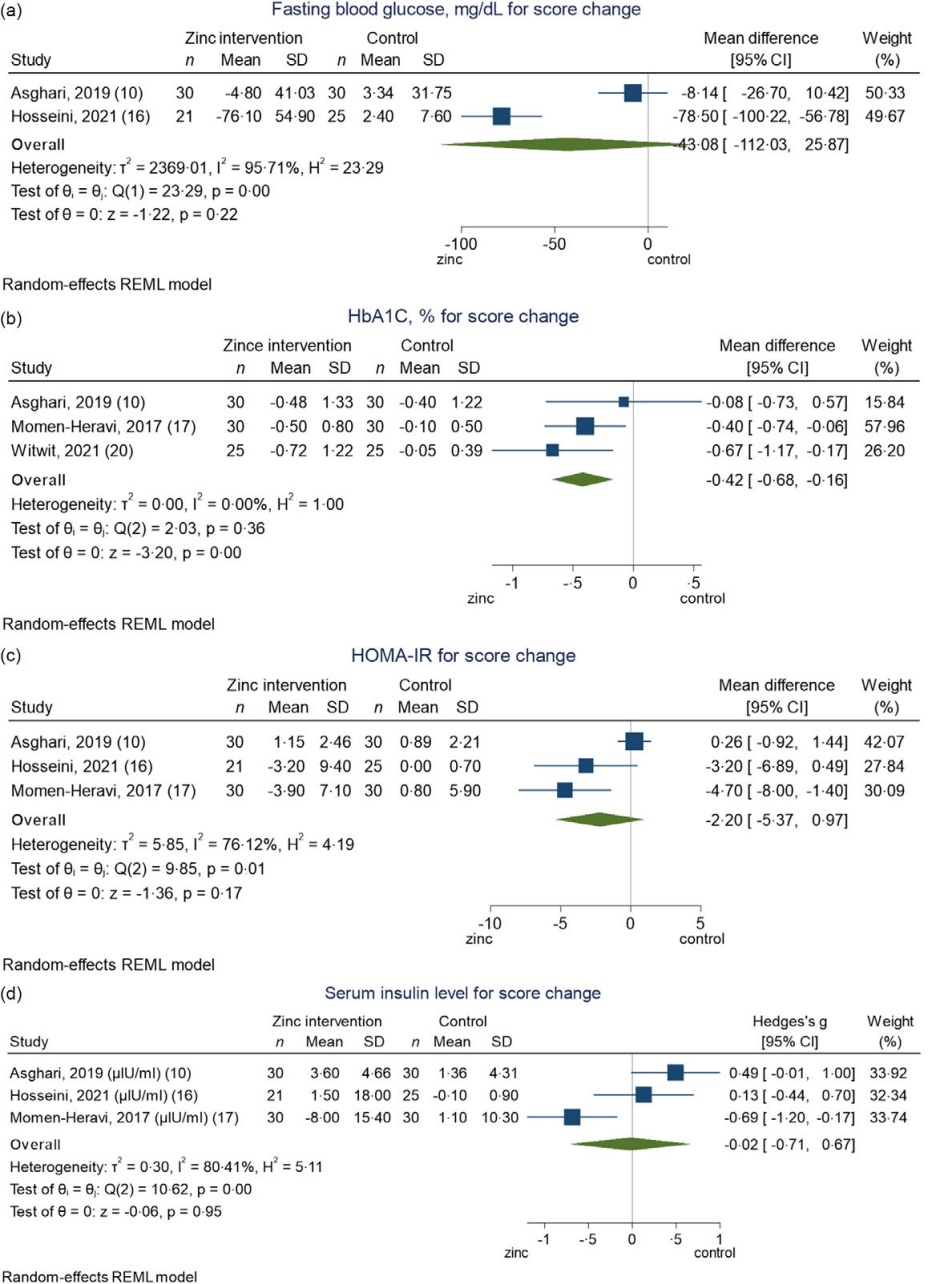
Forest plots summarising the MD or SMD of change scores among fasting blood glucose (a), HbA1c (b), HOMA-IR (c) and serum insulin level (d) between Zn intervention and control arms for low risk-of-bias trials. MD, mean difference; SMD, standardised mean difference; HOMA-IR, homeostatic model assessment for insulin resistance.

The results of the change scores are presented in Fig. 3, but we did not observe any significant results, except with the outcome of HbA1C, which may be because fewer trials reported the measure of change score. The detail of the certainty assessment is presented in Supplementary result 2 and Supplementary Table 2. We did not contact corresponding authors to obtain outcome data if they did not report post-trial means or change scores. Some secondary outcomes were found after full-text reading, and their method and result in *post hoc* analysis are presented in Supplementary Result 3 and Supplementary Fig. 3.

#### Reporting bias assessment

**Fig. 4.**
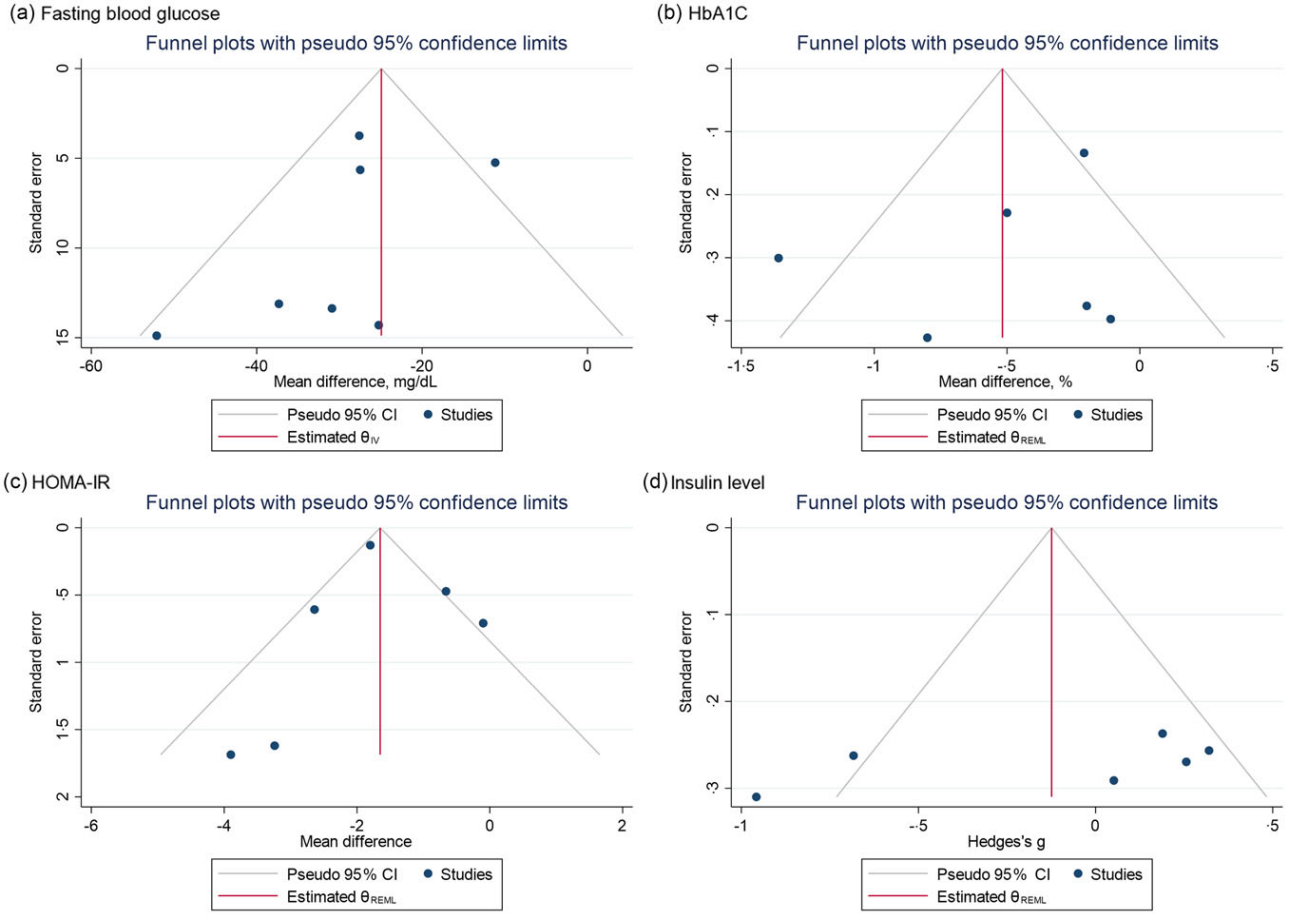
Funnel plots with pseudo 95 % CI demonstrating the MD or SMD of post-trial fasting blood glucose (a), HbA1c (b), HOMA-IR (C) and serum insulin level (d) for each low risk-of-bias trials against their corresponding SEs. MD, mean difference; SMD, standardised mean difference; HOMA-IR, homeostatic model assessment for insulin resistance.

We observed a slight asymmetry in the funnel plot of the fasting blood glucose in Fig. 4(a). One study fell out of the 95 % CI. The *P*-value of the Egger test for small-study effects was 0·1649, which meant there was no strong reporting bias.

Visual assessment of the funnel plots of HbA1c (Fig. 4(b)), HOMA-IR (Fig. 4(c)) and serum insulin level (Fig. 4(d)) suggested that there was no asymmetry. Further, the *P*-value of the Egger test for small-study effects was 0·7341, 0·2697 and 0·1514 for HbA1c, HOMA-IR and serum insulin level, respectively, which fail to reject the null hypothesis of no asymmetry and suggested no significant reporting bias exist.

#### Additional analysis: meta-regression

Second, through meta-regression (regardless of the ROB), we found evidence to support a linear association of fasting blood glucose (regression coefficient: 8·42, 95 % CI (0·52, 16·33)) and HbA1c (regression coefficient: −0·46, 95 % CI (−0·73, −0·19)) with trial duration, which was the significant explanatory variable on heterogeneity. Whether specified diabetes types significantly explained the heterogeneity for the outcome of HOMA-IR (regression coefficient: −1·68, 95 % CI (−2·84, −0·51)) and serum insulin level (regression coefficient: −0·78, 95 % CI (−1·31, −0·25)) in the meta-analyses. The regression coefficient reflected that every level change of explanatory variables would lead to the change of effect size with each outcome’s unit. We shall be cautious about the results because none of the outcomes had more than ten trials. These results are presented in Table 2.

**Table 2.** Meta-regression of potential explanatory variables on heterogeneity in the meta-analysis (Mean values, coefficient values and standard errors)

Outcome	*n* studies	Explanatory factor								
		Dose of Zn intervention			Duration of trial			Diabetes type specified		
		Mean, mg/d	Regression coefficient	95 % CI	Mean, month	Regression coefficient	95 % CI	*n* studies that specified T2D	Regression coefficient	95 % CI
Fasting blood glucose	7	34·75	–0·01	–0·24, 0·23	2·64	8·42	0·52, 16·33*	3	1·31	–18·43, 21·05
HbA1c	6	39·59	0·00	–0·01, 0·01	2·75	–0·46	0·73, −0·19*	4	–0·13	–1·06, 0·77
HOMA-IR	6	20·24	–0·05	–0·11, 0·01	2·67	–0·46	–2·85, 1·93	2	–1·68	2·84, −0·51*
Serum insulin level	6	22·96	–0·00	–0·03, 0·02	2·83	–0·04	–0·73, 0·65	3	–0·78	–1·31, −0·25*

HOMA-IR, homeostatic model assessment for insulin resistance.

* *P* < 0·05.

### Two-sample Mendelian randomisation

#### Description of exposure single-nucleotide polymorphism summary

Three SNP were robustly associated with Zn supplement: rs6756297, rs4861163 and rs10822145, and their GWAS all were conducted from the UK biobank, presented in Table 3. The sample size for the GWAS was 461 384 participants.

**Table 3.** Description of the Zn supplement-SNP association in the two-sample MR (Coefficient values and standard errors)

SNP	Exposure: Zn supplement				
	Sample size	*β*	se	Population	Consortium
rs4861163	461 384	0·006	0·001	European	UK Biobank
rs10822145	461 384	0·002	0·000	European	UK Biobank
rs6756297	461 384	–0·003	0·000	European	UK Biobank

SNP, single-nucleotide polymorphism; MR, Mendelian randomisation.

#### Description of outcome single-nucleotide polymorphism summary

The summary statistics of each outcome with each SNP were reported in Table 4. Since the exposure data was from the UK biobank, we chose the MAGIC consortium with the European population for each outcome.

**Table 4.** Description of the diabetic outcome-SNP association in the two-sample MR (Coefficient values and standard errors)

SNP	Outcome									
	Fasting glucose					HbA1c				
	*n*	*β*	se	Population	Consortium	*n*	*β*	se	Population	Consortium
rs4861163	133 010	0·004	0·002	European	MAGIC	46 368	–0·004	0·004	European	MAGIC
rs10822145	133 010	–0·005	0·002	European	MAGIC	46 368	0·001	0·003	European	MAGIC
rs6756297	58 074	–0·003	0·008	European	MAGIC	46 368	–0·003	0·009	European	MAGIC
	HOMA-IR					Fasting insulin				
rs4861163	37 037	0·004	0·005	European	MAGIC	51 750	0·004	0·003	European	MAGIC
rs10822145	NA	NA	NA	NA	NA	51 750	–0·006	0·004	European	MAGIC
rs6756297	37 037	0·004	0·010	European	MAGIC	51 750	–0·002	0·008	European	MAGIC

SNP, single-nucleotide polymorphism; MR, Mendelian randomisation; HOMA-IR, homeostatic model assessment for insulin resistance.

#### Results of two-sample Mendelian randomisation

**Fig. 5.**
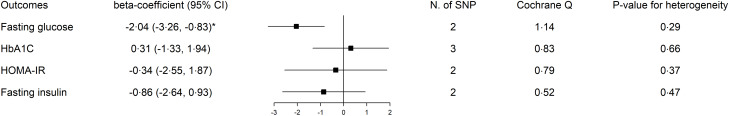
Two-sample MR results with inverse variance weighting coefficient. MR, Mendelian randomisation.

The results including the heterogeneity test are presented in Fig. 5. Zn supplement with two SNP led to a significat decrease in fasting glucose (coefficient: −2·04, 95 % CI (−3·26, −0·83)). The unit of fasting glucose was mmol/l^(67)^ (−2·04 mmol/l = −37·09 mg/dl). While there was no strong evidence supporting the relationship of Zn supplements with HbA1c, HOMA-IR and insulin level (*P* > 0·05). The *P*-value for heterogeneity of fasting glucose, HbA1c, HOMA-IR and insulin level were 0·29, 0·66, 0·37 and 0·47. Neither of these results had significant heterogeneity, which suggested no pleiotropy with the null hypothesis.

## Discussion

### Principal findings

This systematic review, including fourteen RCT between 2003 and 2021, reported how single Zn supplements impacted glycaemic control in T2D. In meta-analyses with random-effect models, Zn supplements were shown to lead to a significant reduction in the level of post-trial fasting blood glucose, HbA1c and HOMA-IR, but not in the serum insulin level. These results had moderate certainty of evidence. Trial duration contributed to the heterogeneity in the results of fasting blood glucose and HbA1c and diabetes type specified contributed to the heterogeneity in HOMA-IR and serum insulin level. In the two-sample MR analysis, with two SNP, Zn supplement was also significantly associated with a lower level of fasting glucose, among the general population. The difference in effect sizes between the review and two-sample MR was small (−26·52 *v*. −37·09, overlapping in CI).

### Previous literature

The protective power of Zn supplements was demonstrated in the previous literature across different populations and diabetes types. In a cohort study encompassing 14 140 Japanese participants, the dietary intake of Zn was associated with a lower OR of T2D mellitus among the younger (age 40–55 years)^(68)^. The result of lower T2D risk was also consistent with an Australian women‘s longitudinal cohort^(69)^. Furthermore, de Carvalho and colleagues found a negative correlation between %HbA1c and plasma Zn levels, and women with deficient Zn levels had higher scores of HOMA-IR and C peptide values in a systematic review^(70)^. Besides, a study with two cohorts observed an 11 % decrease in the risk of gestational hyperglycaemia by every 1 mg/d Zn intake in women, which might be used to prevent gestational diabetes^(71)^.

In 2019, Wang *et al.* indicated that Zn supplementation could significantly reduce key glycaemic indicators in a systematic review of RCT that included all diabetes types. This review involved 1700 participants and found a larger net change among fasting glucose, 2-h postprandial glucose, HbA1c and HOMA-IR in the Zn intervention arm^(21)^. Zn supplements had a larger effect on fasting glucose in diabetes patients than people at high risk and a preventative effect among pre-diabetes^(21)^. These findings were consistent with our results on Zn’s favorable effect on glycaemic control and the magnitude of HbA1c’s MD was similar to ours. But they did not specify the diabetes type in their population included. A systematic review of RCT published in early 2021 specified Zn as a single factor of intervention and found that low-dose and short-duration Zn supplements also showed significant improvements in some T2D indicators, compared with the high dose and long duration^(22)^. The duration-specific effect also supported our result in meta-regression about the heterogeneity contribution of the trial duration, which plays a critical role in the Zn supplement’s effect. However, the true effect of Zn might be masked by the multi-supplement intervention or non-specified diabetes type with differential biological mechanisms.

There are not many MR studies that directly linked Zn as exposure to diabetes. An MR study in 2019 did not find any significant causal relationship between Zn level in blood and odds of T2D with two SNP^(72)^. The difference with our results may be due to the supplementary effect of Zn.

### Strength

To our knowledge, this study is the first to use mixed methods by the systematic review of RCT and MR to triangulate evidence of causal inference in the field of nutritional supplementation. This systematic review included the newest, until 2021, literature with scientific rigor and delivered a clear statement of the Zn supplement’s protection on T2D at the individual level. Our result in improving glycaemic control was consistent with previous literature. Not only that, but we also narrowed the intervention into a single supplement to emphasise the effect of Zn with less concern of contamination bias. Moreover, on the basis of the review, the application of two-sample MR minimised residual confounding or reverse causality due to imperfect randomisation or short duration and gave a more comprehensive lifelong evaluation. MR assumed that the effect of instrumental SNP was only from the exposure of interest to the outcome, independent of other factors. Compared with one-sample MR, two-sample increased statistical power with a larger population from multiple GWAS consortia^(30)^.

### Limitation of included studies, systematic review

The majority of the studies had relatively small groups in each arm (around thirty participants). Although this may limit the power and confounder adjustment, the method of meta-analysis could combine them to get stronger results. Moreover, the trials with at least some concerns of bias were not included in the main interpretation. In the sensitivity analysis which included all trials regardless of the risk of bias, the effect size was similar to the meta-analysis for low-risk only, which reduced our concern (online Supplementary Fig. 2). Suspicious publication bias of fasting blood glucose perhaps came from the small number of trials included. Given the small sample size and variation between trials, the results should be more carefully interpreted and cautiously applied in clinical practice.

Notably, some studies were excluded from the primary analyses for only reporting post-trial means or change scores, which could induce publication bias. For example, Balk et al. analysed the within-group correlation from 123 studies and gave the median correlation values of 0·54 in the treatment group and 0·73 in the control group^(73)^. Using the equation for change scores’ se from the Cochrane handbook^(74)^, we calculated all the possible change scores and their se of HbA1c and performed another sensitivity meta-analysis (online Supplementary Fig. 4). Zn intervention led to a large decrease in HbA1c than the control group. Thus, our initial conclusion appears robust after this sensitivity analysis.

Also, there may exist the problem of generalisability that most of the trials were in Asia, which may induce the problem of extrapolation. A possible explanation is that researchers from high-income countries prefer to perform trials with co-supplement and target a wider population of all types of diabetes. Moreover, inadequate Zn intake was more common in developing countries than in developed countries^(75,76)^. Zn deficiency was also found to aggravate insulin resistance and hyperglycaemia^(77,78)^, therefore researchers from a developing country may prefer to choose Zn supplementary as a single intervention. Moreover, it is possible that the improvement in glycaemic control was due to reducing pre-existing Zn deficiencies within the study population, so that the benefits in the general population may disappear. However, our results were consistent with previous reviews which included studies from developed countries like Australia or USA, which may relieve the problem to some extent^(21,22,79)^. Thus, further RCT with larger sample sizes in a variety of geographic regions would be preferred.

### Limitation of review processes, systematic review

In the review process, the study was limited in that only one reviewer screened every paper and extracted information. The high percentage agreement and Gwet AC in the random set reduced our concerns. Second, we found some degree of heterogeneity in each outcome analysis. Due to the limited number of trials, the meta-regression was not strong enough to detect explanatory variables. We may expand our criteria to include more trials in the situation of not affecting the directness. Moreover, we were aware that the risk of type I error would increase with multiple tests in our study.

### Limitation of two-sample Mendelian randomisation

The MR part was limited to the low number of SNP included. This meant that sensitivity analysis for exclusion restriction assumption and leave-one-out could not be used. Since HbA1c has three SNP in the two-sample MR, the sensitivity analyses were performed, and the results were all non-significant (online Supplementary Table 3). However, MR studies are often low-powered, and the small number of SNP will have additionally reduced the power of the MR analyses, so we did not present other outcomes’ results from the MR-Egger regression, weighted median and weight mode analyses^(80)^. Although there is a potential bias from the effect of supplementary, the negative control using Ca supplementation implied that this is unlikely to have produced a substantive bias (online Supplementary Table 4). The two-sample MR results of negative control: hair colour suggested no significant association between Zn supplementary and hair colour (online Supplementary Table 5), which reduced the threat from bias due to residual population structure. Third, pooled analysis of individual studies made us not access the individual patient data to specify the T2D population.

### Biological plausibility

Zn’s antioxidant properties are the biologically plausible connection to T2D. Oxidative stress reflected an imbalance between the production of reactive oxygen species and antioxidant defenses. Excess reactive oxygen species cause lipid peroxidation, leading to the lesion of cell membranes and lipoproteins, which ultimately damages insulin secretion and increases insulin resistance through the signaling pathway within the *β*-cells of pancreatic islets^(81,82)^. Plasma advance oxidation protein products generated during oxidative stress were indicated as a biomarker of endothelial dysfunction as early events for T2D diabetes^(83)^. As a catalytic role for Zn superoxide dismutase, Zn involves the conversion of superoxide radicals to molecular oxygen and hydrogen peroxide through antagonism, which could reduce reactive oxygen species toxicity^(84,85)^. For chronic effects, Zn is also an inducer of metallothionein in multiple organs^(85)^. The metallothionein immunoreactivity levels were higher in the tubular areas of the Zn-supplemented group, suggesting zinc’s stimulation effect to regulate the oxidative stress in the rat^(86)^. The sulfhydryl stabilisation by Zn protection was another mechanism against oxidative damage^(87)^.

Many studies have investigated the metabolism of Zn in the pathogenesis of diabetes mellitus. In the spontaneously diabetic mice, researchers observed the *in vitro* insulinomimetic activity, hypoglycaemic effect in glucose and insulin resistance attenuation by administration of Zn complexes^(88)^. In the population study, diabetic patients had a higher level of 8-hydroxy-2-deoxyguanosine, oxidative damage to DNA and a lower level of Zn, which suggested more serious oxidative lesions^(89)^. By analogy, people with higher dietary antioxidant capacity (richer antioxidants in diet) were associated with a lower risk of T2D and a lower score of HOMA-IR across sexes in the Rotterdam study^(90)^. Antioxidants become therapeutic options to manage diabetes and its serious complications such as Metformin, which reduces the production of reactive oxygen species and increases insulin sensitivity^(91)^. So, Zn administration has huge potential to be a first-line intervention treatment for T2D due to its antioxidant mechanism.

### Implications

Optimal ways to utilise the Zn supplement intervention in real-world health practices need more concrete explorations in the particular context of disrupted routine care due to the COVID-19 pandemic. According to a rapid WHO assessment, nearly 50 % of diabetes and diabetic complications management services were partially or completely disrupted, which was an essential service in most of countries, especially among low- and middle-income countries^(92)^. Timely nutritional interventions are essential for successful and sustainable diabetes care to overcome the negative effect from the implication of strict infection prevention and control^(93)^. Self-care practice should be encouraged during this extreme health workforce shortage^(94)^. Immediate implementation of Zn as an efficient tool or complement is needed for patients who struggle with limited medical resources and primary care systems, which are considerably disrupted. Nevertheless, we should be discreet about the dose of Zn supplements. According to the Food and Nutrition Board, USA, the recommendations of Zn daily intake for adult males and females are 11 mg and 8 mg^(95)^. Moreover, Gibson *et al.* reviewed the dietary Zn recommendations of multiple international agencies and organisations and suggested around 40 mg/d of tolerable upper intake level of Zn for adults^(96)^. The Zn supplementary suggestion should be given in the consideration of recommended amount and daily diet.

### Conclusion

The study found that a single Zn supplement was strongly associated with a lower level of glycaemic indicators in T2D, suggesting a protective effect. The systematic review and two-sample MR supported a causal association with some evidence but need to be further validated due to fewer studies and SNP included, so the interpretation of the results should be careful. Overall, we advocate the use of Zn supplements, within the proper guideline, as an efficient nutritional intervention to support routine diabetic care.

## Supplementary material

For supplementary material accompanying this paper visit https://doi.org/10.1017/S0007114522002616. click here to view supplementary material

## References

[1] Czech MP (2017) Insulin action and resistance in obesity and type 2 diabetes. Nat Med 23, 804–814. 28697184 10.1038/nm.4350 PMC6048953

[2] Wilcox G (2005) Insulin and insulin resistance. Clin Biochem Rev 26, 19–39. 16278749 PMC1204764

[3] Wexler DJ , Porneala B , Chang Y , et al. (2012) Diabetes differentially affects depression and self-rated health by age in the U.S. Diabetes Care 35, 1575–1577. 22611066 10.2337/dc11-2266 PMC3379579

[4] Yu M , Zhan X , Yang Z , et al. (2021) Measuring the global, regional, and national burden of type 2 diabetes and the attributable risk factors in all 194 countries. J Diabetes 13, 613–639. 33486878 10.1111/1753-0407.13159

[5] Dunachie S & Chamnan P (2019) The double burden of diabetes and global infection in low and middle-income countries. Trans R Soc Trop Med Hyg 113, 56–64. 30517697 10.1093/trstmh/try124 PMC6364794

[6] Joshi SR (2016) Diabetes care in India. Ann Glob Health 81, 830–838. 10.1016/j.aogh.2016.01.002 27108150

[7] Seuring T , Archangelidi O & Suhrcke M (2015) The economic costs of type 2 diabetes: a global systematic review. Pharmacoeconomics 33, 811–831. 25787932 10.1007/s40273-015-0268-9 PMC4519633

[8] Hex N , Bartlett C , Wright D , et al. (2012) Estimating the current and future costs of type 1 and type 2 diabetes in the UK, including direct health costs and indirect societal and productivity costs. Diabet Med 29, 855–862. 22537247 10.1111/j.1464-5491.2012.03698.x

[9] Simon SF & Taylor CG (2001) Dietary zinc supplementation attenuates hyperglycemia in db/db mice. Exp Biol Med 226, 43–51. 10.1177/153537020122600107 11368237

[10] Koerner JD , Vives MJ , O’Connor JP , et al. (2016) Zinc has insulin-mimetic properties which enhance spinal fusion in a rat model. Spine J 16, 777–783. 26850174 10.1016/j.spinee.2016.01.190

[11] Qi S , He J , Zheng H , et al. (2020) Zinc supplementation increased bone mineral density, improves bone histomorphology, and prevents bone loss in diabetic rat. Biol Trace Elem Res 194, 493–501. 31363990 10.1007/s12011-019-01810-7

[12] Norouzi S , Adulcikas J , Sohal SS , et al. (2017) Zinc transporters and insulin resistance: therapeutic implications for type 2 diabetes and metabolic disease. J Biomed Sci 24, 87. 29157234 10.1186/s12929-017-0394-0 PMC5694903

[13] Chabosseau P & Rutter GA (2016) Zinc and Diabetes. 10.1016/j.abb.2016.05.022 (accessed July 2021). 27262257

[14] Manoharan C & Singh J (2015) Addition of zinc improves the physical stability of insulin in the primary emulsification step of the poly(lactide-co-glycolide) microsphere preparation process. Polymers 7, 836–850.

[15] Li YV (2014) Zinc and insulin in pancreatic *β*-cells. Endocrine 45, 178–189. 23979673 10.1007/s12020-013-0032-x

[16] Vincent HK , Bourguignon CM , Weltman AL , et al. (2009) Effects of antioxidant supplementation on insulin sensitivity, endothelial adhesion molecules, and oxidative stress in normal-weight and overweight young adults. Metabolism 58, 254–262. 19154960 10.1016/j.metabol.2008.09.022 PMC3325609

[17] Niki E (1987) Antioxidants in relation to lipid peroxidation. Chem Phys Lipids 44, 227–253. 3311418 10.1016/0009-3084(87)90052-1

[18] Cruz KJC , de Oliveira ARS & Marreiro DN (2015) Antioxidant role of zinc in diabetes mellitus. World J Diabetes 6, 333–337. 25789115 10.4239/wjd.v6.i2.333 PMC4360427

[19] Derosa G , D’Angelo A , Romano D , et al. (2016) A clinical trial about a food supplement containing *α*-lipoic acid on oxidative stress markers in type 2 diabetic patients. Int J Mol Sci 17, 1802. 27801825 10.3390/ijms17111802 PMC5133803

[20] Islam MR , Attia J , Ali L , et al. (2016) Zinc supplementation for improving glucose handling in pre-diabetes: a double blind randomized placebo controlled pilot study. Diabetes Res Clin Pract 115, 39–46. 27242121 10.1016/j.diabres.2016.03.010

[21] Wang X , Wu W , Zheng W , et al. (2019) Zinc supplementation improves glycemic control for diabetes prevention and management: a systematic review and meta-analysis of randomized controlled trials. Am J Clin Nutr 110, 76–90. 31161192 10.1093/ajcn/nqz041

[22] Pompano LM & Boy E (2021) Effects of dose and duration of zinc interventions on risk factors for type 2 diabetes and cardiovascular disease: a systematic review and meta-analysis. Adv Nutr 12, 141–160. 32722790 10.1093/advances/nmaa087 PMC7850144

[23] Melse-Boonstra A (2020) Bioavailability of micronutrients from nutrient-dense whole foods: zooming in on dairy, vegetables, and fruits. Front Nutr 7, 101. 32793622 10.3389/fnut.2020.00101 PMC7393990

[24] Jayalakshmi S & Platel K (2016) Supplemental levels of iron and calcium interfere with repletion of zinc status in zinc-deficient animals. Food Funct 7, 2288–2293. 27101872 10.1039/c6fo00134c

[25] Lawlor DA , Harbord RM , Sterne JAC , et al. (2008) Mendelian randomization: using genes as instruments for making causal inferences in epidemiology. Stat Med 27, 1133–1163. 17886233 10.1002/sim.3034

[26] Davey Smith G & Hemani G (2014) Mendelian randomization: genetic anchors for causal inference in epidemiological studies. Hum Mol Genet 23, R89–R98. 25064373 10.1093/hmg/ddu328 PMC4170722

[27] Evans DM & Davey Smith G (2015) Mendelian randomization: new applications in the coming age of hypothesis-free causality. Annu Rev Genomics Hum Genet 16, 327–350. 25939054 10.1146/annurev-genom-090314-050016

[28] Davies NM , Holmes MV & Davey Smith G (2018) Reading Mendelian randomisation studies: a guide, glossary, and checklist for clinicians. BMJ 362, k601. 30002074 10.1136/bmj.k601 PMC6041728

[29] Davey Smith G & Ebrahim S (2003) ‘Mendelian randomization’: can genetic epidemiology contribute to understanding environmental determinants of disease? Int J Epidemiol 32, 1–22. 12689998 10.1093/ije/dyg070

[30] Lawlor DA (2016) Commentary: two-sample Mendelian randomization: opportunities and challenges. Int J Epidemiol 45, 908–915. 27427429 10.1093/ije/dyw127 PMC5005949

[31] Gibney MJ , McNulty BA , Ryan MF , et al. (2014) Nutritional phenotype databases and integrated nutrition: from molecules to populations. Adv Nutr 5, 352S–357S. 24829488 10.3945/an.113.005496 PMC4013193

[32] Mohammadi-Shemirani P , Chong M , Pigeyre M , et al. (2020) Effects of lifelong testosterone exposure on health and disease using Mendelian randomization. Elife 9, e58914. 33063668 10.7554/eLife.58914 PMC7591257

[33] Yarmolinsky J , Wade KH , Richmond RC , et al. (2018) Causal inference in cancer epidemiology: what is the role of mendelian randomization? Cancer Epidemiol Biomarkers Prev 27, 995–1010. 29941659 10.1158/1055-9965.EPI-17-1177 PMC6522350

[34] Moher D , Liberati A , Tetzlaff J , et al. (2009) Preferred reporting items for systematic reviews and meta-analyses: the PRISMA statement. BMJ 339, b2535. 19622551 10.1136/bmj.b2535 PMC2714657

[35] Gwet KL (2008) Computing inter-rater reliability and its variance in the presence of high agreement. Br J Math Stat Psychol 61, 29–48. 18482474 10.1348/000711006X126600

[36] Data Extraction Forms (2014) Cochrane Developmental, Psychosocial and Learning Problems. https://dplp.cochrane.org/data-extraction-forms (accessed July 2021).

[37] Deeks JJ , Higgins JPT , Altman DG , et al. (2019) Analysing data and undertaking meta-analyses. In Cochrane Handbook for Systematic Reviews of Interventions, pp. 241–284 [ JPT Higgins , J Thomas , J Chandler , et al., editors]. Chichester: Wiley.

[38] Brown J , Ceysens G & Boulvain M (2017) Exercise for pregnant women with gestational diabetes for improving maternal and fetal outcomes. Cochrane Database Syst Rev 6, CD012202. 28639706 10.1002/14651858.CD012202.pub2 PMC6481507

[39] Sterne JAC & Harbord RM (2004) Funnel plots in meta-analysis. Stata J 4, 127–141.

[40] Harbord RM , Harris RJ & Sterne JAC (2009) Updated tests for small-study effects in meta-analyses. Stata J 9, 197–210.

[41] Thompson SG & Higgins JPT (2002) How should meta-regression analyses be undertaken and interpreted? Stat Med 21, 1559–1573. 12111920 10.1002/sim.1187

[42] Skrivankova VW , Richmond RC , Woolf BAR , et al. (2021) Strengthening the reporting of observational studies in epidemiology using mendelian randomisation (STROBE-MR): explanation and elaboration. BMJ 375, n2233. 34702754 10.1136/bmj.n2233 PMC8546498

[43] Hemani G , Zheng J , Elsworth B , et al. (2018) The MR-Base platform supports systematic causal inference across the human phenome. Elife 7, e34408. 29846171 10.7554/eLife.34408 PMC5976434

[44] Bycroft C , Freeman C , Petkova D , et al. (2018) The UK Biobank resource with deep phenotyping and genomic data. Nature 562, 203–209. 30305743 10.1038/s41586-018-0579-z PMC6786975

[45] Silverwood RJ , Holmes MV , Dale CE , et al. (2014) Testing for non-linear causal effects using a binary genotype in a Mendelian randomization study: application to alcohol and cardiovascular traits. Int J Epidemiol 43, 1781–1790. 25192829 10.1093/ije/dyu187 PMC4276061

[46] Mitchell R , Hemani G , Dudding T , et al. (2019): MRC IEU UK Biobank GWAS Pipeline Version 2. 10.5523/bris.pnoat8cxo0u52p6ynfaekeigi (accessed July 2021).

[47] Smith GD & Ebrahim S (2008) Mendelian Randomization: Genetic Variants as Instruments for Strengthening Causal Inference in Observational Studies. https://www.ncbi.nlm.nih.gov/books/NBK62433/ (accessed July 2021).

[48] Hartwig FP , Davies NM , Hemani G , et al. (2016) Two-sample Mendelian randomization: avoiding the downsides of a powerful, widely applicable but potentially fallible technique. Int J Epidemiol 45, 1717–1726. 28338968 10.1093/ije/dyx028 PMC5722032

[49] Walker VM , Davies NM , Hemani G , et al. (2019) Using the MR-Base platform to investigate risk factors and drug targets for thousands of phenotypes. Wellcome Open Res 4, 113. 31448343 10.12688/wellcomeopenres.15334.1 PMC6694718

[50] Bowden J , Hemani G & Davey Smith G (2018) Invited commentary: detecting individual and global horizontal pleiotropy in mendelian randomization – a job for the humble heterogeneity statistic? Am J Epidemiol 187, 2681–2685. 30188969 10.1093/aje/kwy185 PMC6269239

[51] Hemani G , Tilling K & Davey Smith G (2017) Orienting the causal relationship between imprecisely measured traits using GWAS summary data. PLoS Genet 13, e1007081. 29149188 10.1371/journal.pgen.1007081 PMC5711033

[52] Afkhami-Ardekani M , Karimi M , Mohammadi SM , et al. (2008) Effect of zinc sulfate supplementation on lipid and glucose in type 2 diabetic patients. Pakistan J Nutr 7, 550–553.

[53] Asghari S , Hosseinzadeh-Attar MJ , Alipoor E , et al. (2019) Effects of zinc supplementation on serum adiponectin concentration and glycemic control in patients with type 2 diabetes. J Trace Elem Med Biol 55, 20–25. 31345359 10.1016/j.jtemb.2019.05.007

[54] Burki ZG , Hussain M , Burki S , et al. (2017) Effect of zinc supplementation on serum fasting blood sugar and HbA1c in adult diabetics on oral hypoglycemic agents. Gomal J Med Sci 15, 8–11.

[55] Gunasekara P , Hettiarachchi M , Liyanage C , et al. (2011) Effects of zinc and multimineral vitamin supplementation on glycemic and lipid control in adult diabetes. Diabetes Metab Syndr Obes Targets Ther 4, 53–60. 10.2147/DMSO.S16691 PMC3064411 21448322

[56] Hosseini R , Montazerifar F , Shahraki E , et al. (2021) The effects of zinc sulfate supplementation on serum copeptin, C-reactive protein and metabolic markers in zinc-deficient diabetic patients on hemodialysis: a randomized, double-blind, placebo-controlled trial. Biol Trace Elem Res 200, 76–83. 33655432 10.1007/s12011-021-02649-7

[57] Khan MI , Siddique KU , Ashfaq F , et al. (2013) Effect of high-dose zinc supplementation with oral hypoglycemic agents on glycemic control and inflammation in type-2 diabetic nephropathy patients. J Nat Sci Biol Med 4, 336–340. 24082728 10.4103/0976-9668.117002 PMC3783776

[58] Matter RM , Elbarbary NS , Ismail EAR , et al. (2020) Zinc supplementation improves glucose homeostasis in patients with *β*-thalassemia major complicated with diabetes mellitus: a randomized controlled trial. Nutrition 73, 110702. 32007694 10.1016/j.nut.2019.110702

[59] Momen-Heravi M , Barahimi E , Razzaghi R , et al. (2017) The effects of zinc supplementation on wound healing and metabolic status in patients with diabetic foot ulcer: a randomized, double-blind, placebo-controlled trial. Wound Repair Regen 25, 512–520. 28395131 10.1111/wrr.12537

[60] Naghizadeh S , Kheirouri S , Ojaghi H , et al. (2018) Zinc supplementation attenuate diabetic indices in patients with diabetic retinopathy. Prog Nutr 20, 263–269.

[61] Nazem MR , Asadi M , Jabbari N , et al. (2019) Effects of zinc supplementation on superoxide dismutase activity and gene expression, and metabolic parameters in overweight type 2 diabetes patients: a randomized, double-blind, controlled trial. Clin Biochem 69, 15–20. 31129183 10.1016/j.clinbiochem.2019.05.008

[62] Parham M , Amini M , Aminorroaya A , et al. (2008) Effect of zinc supplementation on microalbuminuria in patients with type 2 diabetes: a double blind, randomized, placebo-controlled, cross-over trial. Rev Diabet Stud 5, 102–109. 18795212 10.1900/RDS.2008.5.102 PMC2556442

[63] Heidarian E , Amini M , Parham M , et al. (2009) Effect of zinc supplementation on serum homocysteine in type 2 diabetic patients with microalbuminuria. Rev Diabet Stud 6, 64–70. 19557297 10.1900/RDS.2009.6.64 PMC2712914

[64] Pérez A , Rojas P , Carrasco F , et al. (2018) Zinc supplementation does not affect glucagon response to intravenous glucose and insulin infusion in patients with well-controlled type 2 diabetes. Biol Trace Elem Res 185, 255–261. 29374382 10.1007/s12011-018-1249-6

[65] Roussel AM , Kerkeni A , Zouari N , et al. (2003) Antioxidant effects of zinc supplementation in tunisians with type 2 diabetes mellitus. J Am Coll Nutr 22, 316–321. 12897047 10.1080/07315724.2003.10719310

[66] Witwit GT , Ali BM , Alsaffar Y , et al. (2021) Effect of zinc supplementation on insulin resistance, lipid profile, bmi in type II diabetic patients. Indian J Forensic Med Toxicol 15, 1487–1493.

[67] Scott RA , Lagou V , Welch RP , et al. (2012) Large-scale association analyses identify new loci influencing glycemic traits and provide insight into the underlying biological pathways. Nat Genet 44, 991–1005. 22885924 10.1038/ng.2385 PMC3433394

[68] Eshak ES , Iso H , Maruyama K , et al. (2018) Associations between dietary intakes of iron, copper and zinc with risk of type 2 diabetes mellitus: a large population-based prospective cohort study. Clin Nutr 37, 667–674. 28285974 10.1016/j.clnu.2017.02.010

[69] Vashum KP , McEvoy M , Shi Z , et al. (2013) Is dietary zinc protective for type 2 diabetes? Results from the Australian longitudinal study on women’s health. BMC Endocr Disord 13, 40. 24093747 10.1186/1472-6823-13-40 PMC4015935

[70] de Carvalho GB , Brandão-Lima PN , Maia CSC , et al. (2017) Zinc’s role in the glycemic control of patients with type 2 diabetes: a systematic review. BioMetals 30, 151–162. 28138861 10.1007/s10534-017-9996-y

[71] Bo S , Lezo A , Menato G , et al. (2005) Gestational hyperglycemia, zinc, selenium, and antioxidant vitamins. Nutrition 21, 186–191. 15723747 10.1016/j.nut.2004.05.022

[72] Cheng W-W , Zhu Q & Zhang H-Y (2019) Mineral nutrition and the risk of chronic diseases: a mendelian randomization study. Nutrients 11, 378. 30759836 10.3390/nu11020378 PMC6412267

[73] Balk EM , Earley A , Patel K , et al. (2012) Empirical Assessment of Within-Arm Correlation Imputation in Trials of Continuous Outcomes. Rockville, MD: Agency for Healthcare Research and Quality (US). 23326900

[74] Higgins JPT , Li T & Deeks JJ (2019) Choosing effect measures and computing estimates of effect. In Cochrane Handbook for Systematic Reviews of Interventions, pp. 143–176 [ JPT Higgins , J Thomas , J Chandler , et al., editors]. Chichester: Wiley.

[75] Gupta S , Brazier AKM & Lowe NM (2020) Zinc deficiency in low- and middle-income countries: prevalence and approaches for mitigation. J Hum Nutr Diet 33, 624–643. 32627912 10.1111/jhn.12791

[76] Wessells KR & Brown KH (2012) Estimating the global prevalence of zinc deficiency: results based on zinc availability in national food supplies and the prevalence of stunting. PLOS ONE 7, e50568. 23209782 10.1371/journal.pone.0050568 PMC3510072

[77] Jurowski K , Szewczyk B , Nowak G , et al. (2014) Biological consequences of zinc deficiency in the pathomechanisms of selected diseases. J Biol Inorg Chem 19, 1069–1079. 24748223 10.1007/s00775-014-1139-0 PMC4175048

[78] Hussein M , Fathy W , Hassan A , et al. (2021) Zinc deficiency correlates with severity of diabetic polyneuropathy. Brain Behav 11, e2349. 34521153 10.1002/brb3.2349 PMC8553312

[79] Jayawardena R , Ranasinghe P , Galappatthy P , et al. (2012) Effects of zinc supplementation on diabetes mellitus: a systematic review and meta-analysis. Diabetol Metab Syndr 4, 13. 22515411 10.1186/1758-5996-4-13 PMC3407731

[80] Brion M-JA , Shakhbazov K & Visscher PM (2013) Calculating statistical power in Mendelian randomization studies. Int J Epidemiol 42, 1497–1501. 24159078 10.1093/ije/dyt179 PMC3807619

[81] Rehman K & Akash MSH (2017) Mechanism of generation of oxidative stress and pathophysiology of type 2 diabetes mellitus: how are they interlinked? J Cell Biochem 118, 3577–3585. 28460155 10.1002/jcb.26097

[82] Pizzino G , Irrera N , Cucinotta M , et al. (2017 ) Oxidative stress: harms and benefits for human health. Oxid Med Cell Longev 2017, 8416763. 28819546 10.1155/2017/8416763 PMC5551541

[83] Liang M , Wang J , Xie C , et al. (2014) Increased plasma advanced oxidation protein products is an early marker of endothelial dysfunction in type 2 diabetes patients without albuminuria. J Diabetes 6, 417–426. 24506465 10.1111/1753-0407.12134

[84] Tainer JA , Getzoff ED , Richardson JS , et al. (1983) Structure and mechanism of copper, zinc superoxide dismutase. Nature 306, 284–287. 6316150 10.1038/306284a0

[85] Powell SR (2000) The antioxidant properties of zinc. J Nutr 130, 1447S–1454S. 10801958 10.1093/jn/130.5.1447S

[86] Özcelik D , Nazıroglu M , Tunçdemir M , et al. (2012) Zinc supplementation attenuates metallothionein and oxidative stress changes in kidney of streptozotocin-induced diabetic rats. Biol Trace Elem Res 150, 342–349. 23054862 10.1007/s12011-012-9508-4

[87] Lee SR (2018) Critical role of zinc as either an antioxidant or a prooxidant in cellular systems. Oxid Med Cell Longev 2018, 9156285. 29743987 10.1155/2018/9156285 PMC5884210

[88] Yoshikawa Y , Adachi Y , Yasui H , et al. (2011) Oral administration of bis(aspirinato)zinc(II) complex ameliorates hyperglycemia and metabolic syndrome-like disorders in spontaneously diabetic KK-A^y^ mice: structure–activity relationship on zinc–salicylate complexes. Chem Pharm Bull 59, 972–977. 10.1248/cpb.59.972 21804241

[89] Mahmoud HM , Ali AF & Al-Timimi DJ (2021) Relationship between zinc status and DNA oxidative damage in patients with type 2 diabetes mellitus. Biol Trace Elem Res 199, 1276–1279. 32666431 10.1007/s12011-020-02267-9

[90] van der Schaft N , Schoufour JD , Nano J , et al. (2019) Dietary antioxidant capacity and risk of type 2 diabetes mellitus, prediabetes and insulin resistance: the Rotterdam study. Eur J Epidemiol 34, 853–861. 31399939 10.1007/s10654-019-00548-9 PMC6759671

[91] Teodoro JS , Nunes S , Rolo AP , et al. (2019) Therapeutic options targeting oxidative stress, mitochondrial dysfunction and inflammation to hinder the progression of vascular complications of diabetes. Front Physiol 9, 1857. 30705633 10.3389/fphys.2018.01857 PMC6344610

[92] Wold Health Organization (2020) The Impact of the COVID-19 Pandemic on Noncommunicable Disease Resources and Services: Results of a Rapid Assessment. Geneva: World Health Organization.

[93] Oni T , Micklesfield LK , Wadende P , et al. (2020) Implications of COVID-19 control measures for diet and physical activity, and lessons for addressing other pandemics facing rapidly urbanising countries. Glob Health Action 13, 1810415. 32867606 10.1080/16549716.2020.1810415 PMC7480567

[94] Yadav UN , Rayamajhee B , Mistry SK , et al. (2020) A syndemic perspective on the management of non-communicable diseases amid the COVID-19 pandemic in low- and middle-income countries. Front Public Health 8, 508. 33102414 10.3389/fpubh.2020.00508 PMC7545493

[95] Institue of Medicine (2001) Dietary Reference Intakes for Vitamin A, Vitamin K, Arsenic, Boron, Chromium, Copper, Iodine, Iron, Manganese, Molybdenum, Nickel, Silicon, Vanadium, and Zinc. Washington, DC: The National Academies Press. 25057538

[96] Gibson RS , King JC & Lowe N (2016) A review of dietary zinc recommendations. Food Nutr Bull 37, 443–460. 27312357 10.1177/0379572116652252

