# Supplementary material for: Triangulating evidence for the causal impact of single-intervention zinc supplement on glycaemic control for type 2 diabetes: systematic review and meta-analysis of randomised controlled trial and two-sample Mendelian randomisation
Source: Br J Nutr. 2022 Aug 10;129(11):1929–44. doi: 10.1017/S0007114522002616 (PMC10167665; doi:10.1017/S0007114522002616)
Supplement: Supplementary file 1 [file S0007114522002616sup001.docx]

**Title:** Triangulating evidence for the causal impact of single-intervention zinc supplement on glycemic control for type-2 diabetes: systematic review and meta-analysis of RCT and two-sample Mendelian randomization

**Zhiyang Wang et al. – Online Supplementary Material**

**Content of supplementary material**

- Supplementary method 1: Searching strategy for each database
- Supplementary result 1: Risk-of-bias assessment
- Supplementary result 2: Certainty assessment
- Supplementary result 3: Post-hoc analysis of other outcomes
- Supplementary table 1: Assessment and supporting evidence of risk-of-bias in each study
- Supplementary table 2: GRADE evaluation of each outcome`s certainty
- Supplementary table 3: Sensitivity analysis of two-sample MR between zinc supplementary and HbA1c
- Supplementary table 4: Association between calcium supplementary and diabetic outcomes in the two-sample MR
- Supplementary table 5: Association between zinc supplementary and hair color in the two-sample MR
- Supplementary figure 1: Risk of bias summary figure illustrating assessment about each risk of bias item for each included study based on the revised Cochrane ROB2
- Supplementary figure 2: Forest plots summarizing the MD or SMD of 2h-postprandial blood sugar (A) and fasting plasma glucose (B) between zinc intervention and control arms
- Supplementary figure 3: Forest plots summarizing the MD or SMD of post-trial fasting blood glucose (A), HbA1c (B), HOMA-IR (C), and serum insulin level (D) between zinc intervention and control arm for all trials
- Supplementary figure 4: Forest plot summarizing the MD of change score (calculated and reported) among HbA1c between zinc intervention and control arm for all trials

**Supplementary method 1: Searching strategy for each database**

**PubMed:**

((zinc[MeSH Terms]) OR (zinc[Title/Abstract])) AND ((diabet*[Title/Abstract]) OR (DM[Title/Abstract]) OR (T2M[Title/Abstract]) OR (Diabetes Mellitus[MeSH Terms])) AND ((“blood sugar”[Title/Abstract]) OR (“blood glucose”[Title/Abstract]) OR (glycemic[Title/Abstract]) OR (hyperglycemi*[Title/Abstract]) OR (blood glucose[MeSH Terms]) OR (HbA1c[Title/Abstract]) OR (glycemic control[MeSH Terms])) AND ((control* [Title/Abstract]) OR (blind* [Title/Abstract]) OR (random*[Title/Abstract]) OR (trial*[Title/Abstract]) OR (RCT[Title/Abstract]) OR (placebo*[Title/Abstract]) OR (randomized controlled trial[MeSH Terms]))

**CINAHL Plus:**

(AB zinc OR MH zinc) AND (AB diabet* OR AB DM OR AB T2M OR MH Diabetes Mellitus) AND (AB “blood sugar” OR AB “blood glucose” OR AB glycemic OR AB hyperglycemi* OR AB HbA1c OR MH glycemic control OR MH blood glucose) AND (AB control* OR AB blind* OR AB random* OR AB trial* OR AB RCT* OR AB placebo* OR MH randomized controlled trial)

**EMBASE:**

1 zinc.ab,ti.

2 "diabet*".ab,ti.

3 DM.ab,ti.

4 T2M.ab,ti.

5 blood glucose.ab,ti.

6 blood sugar.ab,ti.

7 glycemic.ab,ti.

8 "hyperglycemi*".ab,ti.

9 HbA1c

10 "control*".ab,ti.

11 "blind*".ab,ti.

12 "random*".ab,ti.

13 "trial*".ab,ti.

14 "RCT*".ab,ti.

15 "placebo*".ab,ti.

16 2 or 3 or 4

17 5 or 6 or 7 or 8 OR 9

18 10 or 11 or 12 or 13 or 14 or 15

19 1 and 16 and 17 and 18

**Web of Science:**

AB=(zinc AND (diabet* OR DM OR T2M ) AND ( “blood sugar” OR “blood glucose” OR glycemic OR hyperglycemi* OR HbA1c) AND (control* OR random* OR trial* OR RCT* OR placebo*) )

**Supplementary result 1: Risk-of-bias assessment**

**Assessment rule**

The Cochrane Risk of Bias tool (ROB 2) was followed as a framework to assess the risk of bias within each study by one unblinded reviewer. It is structured into five domains covered all types of bias that can affect the results of randomized trials:

1. bias arising from the randomization process.
2. bias due to deviations from intended interventions.
3. bias due to missing outcome data.
4. bias in the measurement of the outcome.
5. bias in the selection of the reported result.

The ROB 2 algorithm maps the answer of the signaling question (“no information”, “yes”, “probably yes”, “no”, or “probably no”) to a proposed judgment in each domain and then leads to an overall judgment(1). The level of risk-of-bias is low risk, some concerns, and high risk. The Crossover RCT had an additional domain about carryover and period effect^(1)^.

**Assessment results**

Burki et al.^(2)^ trials raised some concern about the bias arising from the randomization process. Because they lacked information about baseline imbalance. Pérez et al.^(3)^ and Khan et al.^(4)^ had high risk, and Parham et al.^(5)^/Heidarian et al.^(6)^ had some concern on the bias due to missing outcome data. We treated Khan et al.^(4)^ at high risk because some of the participants in the follow-up were excluded from starting insulin use. Pérez et al.^(3)^ was also considered as high risk for further selection criteria for the participant after allocation, which was highly correlated with the effect of interest (HbA1c between 5.7 and 8%). Parham et al.^(5)^/Heidarian et al.^(6)^ had a small group of participants lost due to insulin using, but the crossover design reduced its effect to some extent because the loss was equal in intervention and control arm by self-comparison. Moreover, Roussel et al.^(7)^ demonstrated some concern for bias in the selection of the reported results. Because they did not report the post-trial means for primary outcome outcomes but stated the relation between zinc intervention and outcomes.

All trials did not have the situation in which the zinc administration was carried in the control arm due to some purpose, so the bias risk due to intended intervention deviations was low. All trials used standard and appropriate laboratory assays to measure the outcome, reducing the bias in measurement. Parham et al.^(5)^/Heidarian et al.^(6)^ had similar participants in two sequences, enough washout period and relatively short overall duration, which the risk arising from carryout and crossover effect was low.

In summary, five trials had at least “some concern” level of risk-of-bias. The overall and details of judgment are presented in supplementary table 1 and supplementary figure 1.

**Supplementary result 2: Certainty assessment**

**Assessment rule**

Grading of Recommendations, Assessment, Development, and Evaluation (GRADE) summarizes confidence and rates the certainty of the evidence for the meta-analysis, which takes five considerations: risk of bias, consistency of effect, imprecision, indirectness, and publication bias^(8)^. Nevertheless, the GRADE assessment contained a considerable amount of subjectivity in each decision by necessity^(9)^. There were four levels of the certainty grade: high, moderate, low, and very low.

The consideration of risk-of-bias was regarded as high certainty because we only interpret the results based on meat-analysis of low risk-of-bias studies. In the consideration of consistency, the lower grade was applied to too strong heterogeneity which disturbed the final interpretation^(8)^. In the consideration of imprecision, the optimal information size (OIS) for continuous variables would be calculated. If the included studies did not reach OIS or results’ 95%CI of the meta-analysis were overlarge, I downgrade the evidence certainty in the concern of impression^(8)^. The indirectness’s grading depended on the eligibility criteria of interpreted studies, especially in the comparison of population^(8)^. If participants in studies differed from the review’s proposal, the lower grade would be applied. Our downgrading in publication bias depended on the degree of the funnel plot’s asymmetry and statistical test for small-study effect.

**Assessment decision**

*Risk-of bias*

We removed trials with at least some concern of bias in the main interpretation, so we have high certainty in the consideration of risk-of-bias for all outcomes.

*Imprecision*

We set the type-1 error`s level as 0.05 and the power as 80%. Based on one included trial (Asghari et al. (10)) which calculated the appropriate sample size, we choose the post-trial means in the control and zinc intervention arm (183 and 152.1) and their SE for fasting blood glucose to calculate the OIS. The OIS was 45 participants in each arm. None of the outcomes included studies reached this number. But 95% CIs for each outcome were not overly large, we downgraded to moderate certainty in the consideration of imprecision.

*Inconsistency*

There was some concern of inconsistency between T2D and non-specified diabetes patients for serum insulin level by significant subgroup difference. The heterogeneity seemed not to disturb the pooled estimate, since the result of two subgroups and overall were all insignificant. We downgraded our confidence from high to moderate. Other outcomes did not have strong inconsistency and we used the random-effect model. So that, the rest three outcome have high-level certainty in consideration of inconsistency

*Indirectness*

The indirectness consideration for all four outcomes was downgraded to the moderate level because a part of included studies` population did not specified diabetes type and was with complication, which potentially had a discrepancy with the intended T2D population for the review. We did the subgroup analysis and test the group difference for this concern.

*Publication bias*

For the outcome of fasting blood glucose, we downgraded the consideration of publication bias to a moderate level of quality, since its funnel plot was not very symmetrical. The other outcome`s plots maintained fair symmetry so that they were rated high-level certainty.

Overall, the results of fasting blood glucose, HbA1c, HOMA-IR, and serum insulin level had moderate-level confidence in certainty (supplementary table 2). Because they had a least one consideration was in moderate certainty evidence.

**Supplementary result 3: Post-hoc analysis of other outcomes**

Some secondary outcomes were found after full-text reading. Without considering the risk-of-bias, three trials reported the post-trial mean of 2h-postprandial blood sugar (supplementary figure 3). We found zinc intervention`s 2h-postprandial blood sugar was significantly lower than control arm (SMD: -1.11, 95%CI: -1.71, -0.50), with 3 trials at end of the trial. While there was only weak evidence to indicate a difference in the post-trial fasting plasma glucose between zinc intervention and control arms (MD: -13.54, 95%CI: -48.5, 21.41), with 2 trials. These meta-analyses used the random-effect model.

One study reported a significant decrease in random blood glucose between pre-trial and post-trial in the zinc intervention arm and this decrease was stronger than the change in the control arm (11). Another study indicated that serum homocysteine levels also reduced significantly in the zinc intervention arm. Homocysteine is a homologue of the amino acid cysteine, which was found to correlate with insulin resistance through hyperhomocysteinemia and other metabolic mechanism^(12,13)^.

**Supplementary table 1: Assessment and supporting evidence of risk-of-bias in each study**

| Study ID | Support or Judgement for risk of bias | | | | | | |
| --- | --- | --- | --- | --- | --- | --- | --- |
|  | Randomization process | Deviations from intended interventions | Missing outcome data | Outcome measurement | Selection of the reported results | Carry and crossover effect | Overall |
| Afkhami – Ardekani, 2008^(14)^ | Randomized process and no baseline imbalance (Low) | No deviation found (Low) | Outcome data on all participants (Low) | Clear lab assay indicates (Low) | Reported eligible data (Low) | NA | low risk in all domains (Low) |
| Asghari, 2019^(10)^ | Randomized process and no baseline imbalance (Low) | No deviation found (Low) | Outcome data on all participants (Low) | Clear lab assay indicates (Low) | Reported eligible data (Low) | NA | low risk in all domains (Low) |
| Burki, 2017^(2)^ | Randomized process and unclear baseline (some concern) | No deviation found (Low) | Outcome data on all participants (Low) | Clear lab assay indicates (Low) | Reported eligible data (Low) | NA | Concern in some domain (Some concern) |
| Gunasekara, 2011^(15)^ | Randomized process and no baseline imbalance (Low) | No deviation found (Low) | Outcome data on all participants (Low) | Clear lab assay indicates (Low) | Reported eligible data (Low) | NA | low risk in all domains (Low) |
| Hosseini, 2021^(16)^ | Randomized process and no baseline imbalance (Low) | No deviation found (Low) | Outcome data on all participants (Low) | Clear lab assay indicates (Low) | Reported eligible data (Low) | NA | low risk in all domains (Low) |
| Khan, 2013^(4)^ | Randomized process and no baseline imbalance (Low) | No deviation found (Low) | Lost participants due to start using insulin (High) | Clear lab assay indicates (Low) | Reported eligible data (Low) | NA | High risk in some domain (High) |
| Matter, 2020^(11)^ | Randomized process and no baseline imbalance (Low) | No deviation found (Low) | Outcome data on all participants (Low) | Clear lab assay indicates (Low) | Reported eligible data (Low) | NA | low risk in all domains (Low) |
| Momen-Heravi, 2017^(17)^ | Randomized process and no baseline imbalance (Low) | No deviation found (Low) | Outcome data on all participants (Low) | Clear lab assay indicates (Low) | Reported eligible data (Low) | NA | low risk in all domains (Low) |
| Naghizadeh, 2018^(18)^ | Randomized process and no baseline imbalance (Low) | No deviation found (Low) | Outcome data on all participants (Low) | Clear lab assay indicates (Low) | Reported eligible data (Low) | NA | low risk in all domains (Low) |
| Nazem, 2019^(19)^ | Randomized process and no baseline imbalance (Low) | No deviation found (Low) | Outcome data on all participants (Low) | Clear lab assay indicates (Low) | Reported eligible data (Low) | NA | low risk in all domains (Low) |
| Parham, 2008^(5)^, Heidarian, 2009^(6)^ | Randomized process and no baseline imbalance (Low) | No deviation found (Low) | Lost participants due to start using insulin in both sequences (some concern) | Clear lab assay indicates (Low) | Reported eligible data (Low) | No carry and crossover effect apparent (Low) | Concern in some domain (Some concern) |
| Pérez, 2018^(3)^ | Randomized process and no baseline imbalance (Low) | No deviation found (Low) | Further selection after randomization and criteria correlated with T2D (High) | Clear lab assay indicates (Low) | Reported eligible data (Low) | NA | High risk in some domain (High) |
| Roussel, 2003^(7)^ | Randomized process and no baseline imbalance (Low) | No deviation found (Low) | Outcome data on all participants (Low) | Clear lab assay indicates (Low) | Only report association with zinc but no post-trial mean (some concern) | NA | Concern in some domain (Some concern) |
| Witwit, 2021^(20)^ | Randomized process and no baseline imbalance (Low) | No deviation found (Low) | Outcome data on all participants (Low) | Clear lab assay indicates (Low) | Reported eligible data (Low) | NA | low risk in all domains (Low) |

**Supplementary table 2: GRADE evaluation of each outcome`s certainty**

| Outcomes | Risk of bias | Imprecision | Inconsistency | Indirectness | Publication bias | Overall |
| --- | --- | --- | --- | --- | --- | --- |
| Fasting blood glucose | ⊕⊕⊕⊕  High | ⊕⊕⊕  Moderate | ⊕⊕⊕⊕  High | ⊕⊕⊕  Moderate | ⊕⊕⊕  Moderate | ⊕⊕⊕  Moderate |
| HbA1c | ⊕⊕⊕⊕  High | ⊕⊕⊕  Moderate | ⊕⊕⊕⊕  High | ⊕⊕⊕  Moderate | ⊕⊕⊕⊕  High | ⊕⊕⊕  Moderate |
| HOMA-IR | ⊕⊕⊕⊕  High | ⊕⊕⊕  Moderate | ⊕⊕⊕⊕  High | ⊕⊕⊕  Moderate | ⊕⊕⊕⊕  High | ⊕⊕⊕  Moderate |
| Serum insulin level | ⊕⊕⊕⊕  High | ⊕⊕⊕  Moderate | ⊕⊕⊕  Moderate | ⊕⊕⊕  Moderate | ⊕⊕⊕⊕  High | ⊕⊕⊕  Moderate |

**Supplementary table 3: Sensitivity analysis of two-sample MR between zinc supplementary and HbA1c**

| Outcome: HbA1c | | | |
| --- | --- | --- | --- |
| Method | Coefficient | SD | P-value |
| MR Egger | -1.17 | 2.38 | 0.71 |
| Weighted median | 0.23 | 1.05 | 0.82 |
| Weighted mode | 0.03 | 1.14 | 0.98 |

**Supplementary table 4: Association between calcium supplementary and diabetic outcomes in the two-sample MR**

| Outcome | *n* SNPs | IVW coefficient | P-value |
| --- | --- | --- | --- |
| Fasting glucose | 3 | -0.32 | 0.62 |
| HbA1c | 3 | 0.36 | 0.56 |
| HOMA-IR | 3 | -0.65 | 0.37 |
| Insulin level | 3 | 0.09 | 0.83 |

**Supplementary table 5: Association between zinc supplementary and hair color in the two-sample MR**

| hair color | *n* SNPs | IVW coefficient | P-value |
| --- | --- | --- | --- |
| Blonde | 3 | -0.20 | 0.27 |
| Red | 3 | -0.14 | 0.46 |
| Light brown | 3 | 0.16 | 0.58 |
| Dark brown | 3 | 0.03 | 0.94 |
| Black | 3 | 0.16 | 0.18 |
| other | 3 | 0.00 | 0.95 |

**Supplementary figure 1: Risk of bias summary figure illustrating assessment about each risk of bias item for each included study based on the revised Cochrane ROB2**

**Supplementary figure 2: Forest plots summarizing the MD or SMD of 2h-postprandial blood sugar (A) and fasting plasma glucose (B) between zinc intervention and control arms**

**Supplementary figure 3: Forest plots summarizing the MD or SMD of post-trial fasting blood glucose (A), HbA1c (B), HOMA-IR (C), and serum insulin level (D) between zinc intervention and control arm for all trials**

Note: For fasting blood glucose, Burki 2017 did not provide the post-trial means, only change score. Roussel 2003 only provided baseline mean and no post-trial mean. For HbA1c, Burki 2017 did not provide the post-trial means, only change score. Roussel 2003 only provided baseline mean and no post-trial mean. Pérez 2018 provided quarter 1 and 3 instead of the standard deviation. Witwit 2021 did not provided the standard deviation. For HOMA-IR, Pérez 2018 provided quarter 1 and 3 instead of the standard deviation. For serum insulin level, Roussel 2003 only provided baseline mean and no post-trial mean. Witwit 2021 did not provided the standard deviation.

**Supplementary figure 4:** **Forest plot summarizing the MD of change score (calculated and reported) among HbA1c between zinc intervention and control arm for all trials**

*: The score change was calculated by this equation and correlation value from Balk et al. (21). The correlation value in the intervention group was 0.54 and in the control group is 0.73.

**Supplementary Reference**

1. Sterne JAC, Savović J, Page MJ, et al. RoB 2: a revised tool for assessing risk of bias in randomised trials. BMJ [Internet]. 2019 Aug 28;366:l4898. Available from: http://www.bmj.com/content/366/bmj.l4898.abstract

2. Burki ZG, Hussain M, Burki S, et al. Effect of Zinc Supplementation on serum fasting blood sugar and HbA1c in adult diabetics on oral hypoglycemic agents. Gomal J Med Sci [Internet]. 2017;15(1):8–11. Available from: http://search.ebscohost.com/login.aspx?direct=true&db=a9h&AN=124889699&site=eds-live

3. Pérez A, Rojas P, Carrasco F, et al. Zinc Supplementation Does Not Affect Glucagon Response to Intravenous Glucose and Insulin Infusion in Patients with Well-Controlled Type 2 Diabetes. Biol Trace Elem Res. 2018;185(2):255–61.

4. Khan MI, Siddique KU, Ashfaq F, et al. Effect of high-dose zinc supplementation with oral hypoglycemic agents on glycemic control and inflammation in type-2 diabetic nephropathy patients. J Nat Sci Biol Med [Internet]. 2013 Jul;4(2):336–40. Available from: https://pubmed.ncbi.nlm.nih.gov/24082728

5. Parham M, Amini M, Aminorroaya A, et al. Effect of zinc supplementation on microalbuminuria in patients with type 2 diabetes: A double blind, randomized, placebo-controlled, cross-over trial. Rev Diabet Stud. 2008;5(2):102–9.

6. Heidarian E, Amini M, Parham M, et al. Effect of Zinc Supplementation on Serum Homocysteine in Type 2 Diabetic Patients with Microalbuminuria. Rev Diabet Stud. 2009;6(1):64–70.

7. Roussel AM, Kerkeni A, Zouari N, et al. Antioxidant effects of zinc supplementation in tunisians with type 2 diabetes mellitus. J Am Coll Nutr. 2003;22(4):316–21.

8. Schünemann HJ, Higgins JPT, Vist GE, et al. Completing‘Summary of findings’tables and grading the certainty of the evidence [Internet]. Cochrane Handbook for Systematic Reviews of Interventions. 2019. p. 375–402. (Wiley Online Books). Available from: https://doi.org/10.1002/9781119536604.ch14

9. Meader N, King K, Llewellyn A, et al. A checklist designed to aid consistency and reproducibility of GRADE assessments: development and pilot validation. Syst Rev [Internet]. 2014 Jul 24;3:82. Available from: https://pubmed.ncbi.nlm.nih.gov/25056145

10. Asghari S, Hosseinzadeh-Attar MJ, Alipoor E, et al. Effects of zinc supplementation on serum adiponectin concentration and glycemic control in patients with type 2 diabetes. J Trace Elem Med Biol [Internet]. 2019;55(44):20–5. Available from: https://doi.org/10.1016/j.jtemb.2019.05.007

11. Matter RM, Elbarbary NS, Ismail EAR, et al. Zinc supplementation improves glucose homeostasis in patients with β-thalassemia major complicated with diabetes mellitus: A randomized controlled trial. Nutrition. 2020;73.

12. Meigs JB, Jacques PF, Selhub J, et al. Fasting Plasma Homocysteine Levels in the Insulin Resistance Syndrome. Diabetes Care [Internet]. 2001 Aug 1;24(8):1403 LP – 1410. Available from: http://care.diabetesjournals.org/content/24/8/1403.abstract

13. Yang N, Yao Z, Miao L, et al. Novel Clinical Evidence of an Association between Homocysteine and Insulin Resistance in Patients with Hypothyroidism or Subclinical Hypothyroidism. PLoS One [Internet]. 2015 May 4;10(5):e0125922. Available from: https://doi.org/10.1371/journal.pone.0125922

14. Afkhami-Ardekani M, Karimi M, Mohammadi SM, et al. Effect of zinc sulfate supplementation on lipid and glucose in type 2 diabetic patients. Pakistan J Nutr. 2008;7(4):550–3.

15. Gunasekara P, Hettiarachchi M, Liyanage C, et al. Effects of zinc and multimineral vitamin supplementation on glycemic and lipid control in adult diabetes. Diabetes, Metab Syndr Obes Targets Ther. 2011;4:53–60.

16. Hosseini R, Montazerifar F, Shahraki E, et al. The Effects of Zinc Sulfate Supplementation on Serum Copeptin, C-Reactive Protein and Metabolic Markers in Zinc-Deficient Diabetic Patients on Hemodialysis: A Randomized, Double-Blind, Placebo-Controlled Trial. Biol Trace Elem Res. 2021;

17. Momen-Heravi M, Barahimi E, Razzaghi R, et al. The effects of zinc supplementation on wound healing and metabolic status in patients with diabetic foot ulcer: A randomized, double-blind, placebo-controlled trial. Wound Repair Regen. 2017;25(3):512–20.

18. Naghizadeh S, Kheirouri S, Ojaghi H, et al. Zinc supplementation attenuate diabetic indices in patients with diabetic retinopathy. Prog Nutr. 2018;20(6):263–9.

19. Nazem MR, Asadi M, Jabbari N, et al. Effects of zinc supplementation on superoxide dismutase activity and gene expression, and metabolic parameters in overweight type 2 diabetes patients: A randomized, double-blind, controlled trial. Clin Biochem [Internet]. 2019;69(May):15–20. Available from: https://doi.org/10.1016/j.clinbiochem.2019.05.008

20. Witwit GT, Ali BM, Alsaffar Y, et al. Effect of Zinc Supplementation on Insulin Resistance, Lipid Profile, BMI in Type II Diabetic Patients. Indian J Forensic Med Toxicol. 2021;15(3):1487–93.

21. Balk EM, Earley A, Patel K, et al. Empirical Assessment of Within-Arm Correlation Imputation in Trials of Continuous Outcomes. Methods Res Reports [Internet]. 2012 [cited 2022 Mar 3];12(EHC141):EF. Available from: https://www.ncbi.nlm.nih.gov/books/NBK115797/
